# Spatial uncertainty and environmental geometry in navigation

**DOI:** 10.1101/2023.01.30.526278

**Published:** 2023-02-01

**Authors:** Yul HR Kang, Daniel M Wolpert, Máté Lengyel

**Affiliations:** 1 Computational and Biological Learning Lab, Department of Engineering, University of Cambridge, Cambridge, UK; 2 Department of Biological and Experimental Psychology, Queen Mary University of London, London, UK; 3 Zuckerman Mind Brain Behavior Institute, Columbia University, New York, NY, USA; 4 Department of Neuroscience, Columbia University, New York, NY, USA; 5 Center for Cognitive Computation, Department of Cognitive Science, Central European University, Budapest, Hungary

## Abstract

Variations in the geometry of the environment, such as the shape and size of an enclosure, have profound effects on navigational behavior and its neural underpinning. Here, we show that these effects arise as a consequence of a single, unifying principle: to navigate efficiently, the brain must maintain and update the uncertainty about one’s location. We developed an image-computable Bayesian ideal observer model of navigation, continually combining noisy visual and self-motion inputs, and a neural encoding model optimized to represent the location uncertainty computed by the ideal observer. Through mathematical analysis and numerical simulations, we show that the ideal observer accounts for a diverse range of sometimes paradoxical distortions of human homing behavior in anisotropic and deformed environments, including ‘boundary tethering’, and its neural encoding accounts for distortions of rodent grid cell responses under identical environmental manipulations. Our results demonstrate that spatial uncertainty plays a key role in navigation.

Determining our location in an environment is an essential component of successful navigation^1^. The neural underpinning of this process involves some of the most intensively studied cell types in the brain, the place cells of the hippocampus and the grid cells of the medial entorhinal cortex^2,3^. In particular, grid cells exhibit spatially ordered, periodic patterns of firing that have been hypothesized to provide a regular ‘metric for space’^4^. However, both navigational behavior and regular, grid-like neural responses seem to go awry when the geometry of an enclosure is manipulated. For example, compared to isotropic, square-shaped environments, human spatial memory exhibits particular biases in an anisotropic environment with trapezoidal boundary geometry^5^. Under identical conditions, the tuning curves of grid cells in rats also become less regular and more anisotropic in shape^6^. When the size of the environment undergoes an unexpected change, such that it expands or compresses along an axis, homing behavior and neural responses do not only scale with the environment^7,8^ but also, paradoxically, show changes along the orthogonal, unchanged axis^8–10^. Moreover, spatial patterns of homing behavior and grid cell responses dissociate depending on which wall along the changed axis was last approached^11,12^.

Previous theories explained only a subset of these results, e.g. the effects of either environmental shape^5,13–17^ or changes in size^8,9,11,12^, but not both, or were only able to explain behavioral^9^ or neural data^14–17^. In addition, they explained the apparent anomalies in behavioral response patterns as epiphenomena of deformations in neural response patterns, without addressing the potential adaptive value of these ‘anomalies’ from a normative perspective^5,9,12^. Here we show instead that all these data can be accounted for by a single, unifying principle: that allocentric location is fundamentally uncertain, and this uncertainty thus pervades both behavioral and neural responses. While the importance of representing uncertainty about spatial location is well accepted in engineering solutions to navigation^18,19^ (even mobile phone navigation apps show a simple form of it by the shaded blue disc around the currently estimated location), its role in biological navigation has only rarely been considered^20–25^. In particular, the fundamental effects of environmental geometry on spatial uncertainty have not been studied.

Formalizing the effects of environmental geometry on navigation is challenging because it can affect visual input, a main source of information for navigation, in complex ways that may not be well captured by simple, discrete point-like landmarks as used in some virtual-reality experiments^26–28^ and assumed to provide direct distance and bearing information in related modeling studies^20,26,29^. Thus, we constructed the ‘Bayesian image-computable observer for navigation’ (BION) model, an ideal observer that integrates information from successive retinal images and idiothetic (self-motion) cues to compute and continuously update a posterior probability distribution over pose (location and head direction). The posterior distribution formalizes uncertainty by expressing the probability with which the observer currently believes themselves to be in each pose given past and current sensory information. This ideal observer reproduced empirically observed patterns of homing behavior in spatial memory experiments. By deriving analytical expressions for how uncertainty depends on environmental geometry in the BION model, we were also able to provide intuitions for how the fundamental principles of optics (the retinal projection) and optimal multi-sensory cue combination explain the behavioral data. Encoding the posterior distributions of the ideal observer in neural population responses also allowed us to correctly predict how the tuning curves of entorhinal grid cells deform as a function of environmental geometry, and become tethered to boundaries when those boundaries are moved. These results demonstrate that spatial uncertainty plays a key role in navigation and in its neural underpinning.

## Results

### The BION model

**Fig. 1 |:**
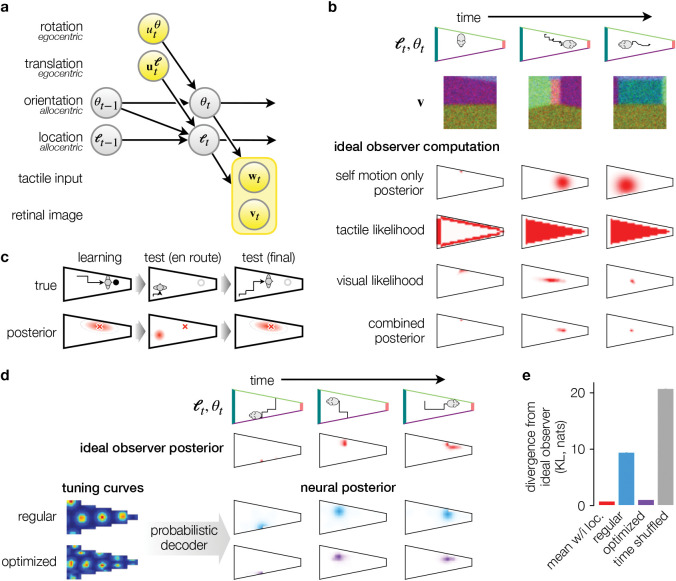
The BION model. **a.** Generative model of navigation. The subject’s allocentric head direction (θ) and location (ℓ) are updated by egocentric self-motion (*u*^*θ*^ and **u**^ℓ^ for rotation and translation, respectively) and give rise to observations of visual input (**v**) and, potentially, tactile input (**w**). Yellow circles denote observed variables, gray circles denote hidden variables. **b.** *Row 1.* Top-down view of a rodent starting from a known location (1^st^ column) and progressing along a trajectory (black line, 2^nd^ and 3^rd^ columns). *Row 2.* First-person view of the 3D environment (**v**) for the corresponding columns of the rodent shown in row 1. The view is corrupted by blur and Poisson noise (reduced for visualization). *Rows 3–6* show ideal observer inferences about location using different inputs (red). *Row 3.* Location posterior based only on self motion, without visual or tactile input, $\text{P}\left(\ell_{t}|u_{0:t}^{\theta },\;u_{0:t}^{\ell }\right)$. *Row 4.* Location likelihood based only on tactile input, P(**w**_*t*_|*ℓ*_*t*_). *Row 5.* Location likelihood based only on visual input, P(**v**_*t*_|ℓ_*t*_). *Row 6.* Location posterior when all inputs are available, $\text{P}\left(\ell_{t}|v_{0:t},\;w_{0:t},\;u_{0:t}^{\theta },\;u_{0:t}^{\ell }\right)$. **c.** Modeling behavior in the ‘homing task’. Top row shows the participant location and bottom row shows the posterior distribution over locations (red shading). Learning the location of an object (black circle, 1^st^ column) is modeled as encoding the mean (red ×) of the posterior in memory. During the test phase, the participant moves from a new location towards the remembered location of the now invisible object (2^nd^ column). Navigational choices decrease the mean squared error between the remembered location of the object and the current location according to the current posterior, until no further decrease is possible (3^rd^ column). **d.** Modeling neural responses. *Rows 1–2*. Location of a rodent exploring a trapezoid environment (*Row 1*) and corresponding ideal observer posteriors (*Row 2*). *Rows 3–4*. Grid cell responses (left) are fed into a probabilistic decoder to compute a neural posterior (right). With a regular grid (*Row 3*) the neural and ideal observer posteriors do not match. When the turning curves are optimized by ‘warping’ (*Row 4*), the neural posterior matches the ideal observer posterior more accurately. **e.** Average (across-time) Kullback-Leibler divergence of ideal observer posteriors from neural posteriors decoded from regular tuning curves (blue) and optimized tuning curves (purple). For reference, the minimal achievable divergence is shown as the divergence of the ideal observer posterior from the average ideal observer posterior at each location (red). Chance level is shown as the divergence between time-shuffled ideal observer posteriors (gray). Error bars show s.e.m. across time points.

In order to study spatial uncertainty, we formalized the task of estimating one’s location in the environment as recursive probabilistic inference given noisy sensory inputs (Fig. 1, Methods). This formalization, based on widely used approaches to robotic navigation^18,19^, treats allocentric pose (location and head direction) as hidden variables to which the brain has no direct access (Fig. 1a). Instead, sensory inputs deliver egocentric signals, such as a first-person view of the environment by visual inputs, and self-motion cues from a variety of senses (e.g. the vestibular system). Thus, the BION model infers allocentric pose from a stream of egocentric sensory inputs based on an internal model for how self-motion cues about translation and rotation (Fig. 1a, **u**^*ℓ*^ and *u*^*θ*^ ) provide information about changes in pose (Fig. 1a, *ℓ* and *θ* for location and head direction, respectively), and how the retinal image depends on the current pose (Fig. 1a, **v**). As we expected environmental geometry to have complex effects on visual information that may not be well described by abstract Lidar-like readings of distance and bearing to point-like landmarks that were often used previously^20,26,29,30^, we adopted an image-computable approach instead^31^. Thus, we considered a fully rendered retinal image through a pinhole projection (with Gaussian blur and Poisson noise). When modeling rodent navigation (but not human navigation in virtual reality), we also considered tactile input, providing egocentric information about whether the animal is next to a wall, and if so, on which side of the animal the wall is (Fig. 1a, **w**).

As the animal moves around in the environment (Fig. 1b, row 1), it receives a sequence of sensory inputs (Fig. 1b, row 2 shows visual input). Given the sequence of inputs received thus far, the ideal observer computes a posterior distribution, expressing the probability with which it currently believes itself to be at each location (and head direction, not shown; Fig. 1b, rows 3–6). When the ideal observer only has access to self-motion inputs, this results in the well-known inevitable accumulation of errors over time by pure path integration (Fig. 1b, row 3). Tactile inputs provide information about being somewhere near the walls, but leave high levels of uncertainty when the animal is away from the walls (Fig. 1b, row 4). Compared to other senses, visual input can result in particularly complex and non-monotonic changes in uncertainty, especially along the line of sight, depending on the current pose of the animal relative to the geometry of the environment (Fig. 1b, row 5). Computing the Bayesian posterior with self-motion, tactile, and visual inputs allows the ideal observer to perform optimal multi-sensory cue combination. In particular, the posterior (Fig. 1b, row 6) is more precise than what each sensory modality alone can afford.

We used the BION model to make predictions about both behavioral patterns in human spatial memory experiments and grid cell recordings in rodents (Methods). To be able to maximally constrain the model, we selected a set of paradigms for which both behavioral and neural data was available. In addition, we considered experiments in which participants had already been familiarized with an environment so that they had minimal uncertainty about its layout (even though in the test phase of some experiments, the actual layout was changed unbeknownst to them). This allowed us to ignore uncertainty about the ‘map’ (see e.g. Ref. 20) and focus on the effects of uncertainty about location with respect to the map.

The behavioral paradigms we modeled required human participants to perform a ‘homing task’, i.e. to return to the location of a specific object whose location they had to previously encode in memory. These experiments use virtual reality environments so that visual input can be precisely controlled. We modeled both the learning and the test phases of these experiments (Fig. 1c). In the learning phase, we simulated trajectories that led participants to the object locations that they needed to remember and, at the end of each such trajectory, stored in memory the mean of the posterior distribution over locations (with noise to account for memory decay) as the remembered location (Fig. 1c, left). In the test phase, participants were removed from the original location of the object, and we modeled their sequence of navigational choices as greedily (albeit noisily) decreasing the mean squared error between their current location and the remembered location of the target object, until no further decrease was possible (Fig. 1c, center and right). Critically, due to the bias-variance trade-off inherent in minimizing the mean squared error^32^, the expected distance was generally not minimized simply where the mean of the current location posterior matched the remembered target location, and thus participants’ uncertainty (the covariance of their posterior) also affected the end point of their chosen routes.

For predicting grid cell responses, we simulated a population of grid cells (Fig. 1d, bottom left) in a randomly foraging animal (Fig. 1d, row 1), and used a probabilistic population decoder^33^ to compute a ‘neural’ posterior over locations from their responses (Fig. 1d, right; Methods). In contrast to standard approaches to efficient coding that aim to minimize the uncertainty in the neural posterior^34^, we required the uncertainty of the neural posterior to faithfully reflect that of the ideal observer (Fig. 1d, row 2), which prescribes the normatively appropriate level of uncertainty given noise and ambiguity in sensory inputs.

In general, the ideal observer’s posterior under-went dynamic changes over time (cf. Fig. 1b, row 6), such that it expressed less or more uncertainty about the animal’s location (Fig. 1d, row 2, e.g. compare steps 1 and 2), sometimes being more isotropic, expressing approximately the same amount of uncertainty along each direction, while at other times becoming anisotropic, i.e. strongly elongated along a particular direction (compare time steps 2 and 3). Critically, the neural posteriors for grid cell responses with perfect hexagonal symmetry were translation-invariant and isotropic (except for trivial boundary effects), failing to exhibit the modulations in the uncertainty the ideal observer expressed (Fig. 1d, row 3). We thus created a ‘warped population code’^16,34^ by warping the underlying grid on which grid cell tuning curves were defined. Specifically, we optimized this warping until the resulting neural posteriors maximally matched the ideal observer posteriors moment-by-moment (Fig. 1d, row 4 and Fig. 1e). The simulated grid cell responses obtained with the optimized warping constituted our neural predictions. Thus, rather than building a theory for the basic hexagonal symmetry of grid fields, as in most previous approaches^35–41^, we focused on modeling how and why this perfect symmetry is broken under well-controlled experimental manipulations.

### Deformations arise from inhomogeneous uncertainty

**Fig. 2 |:**
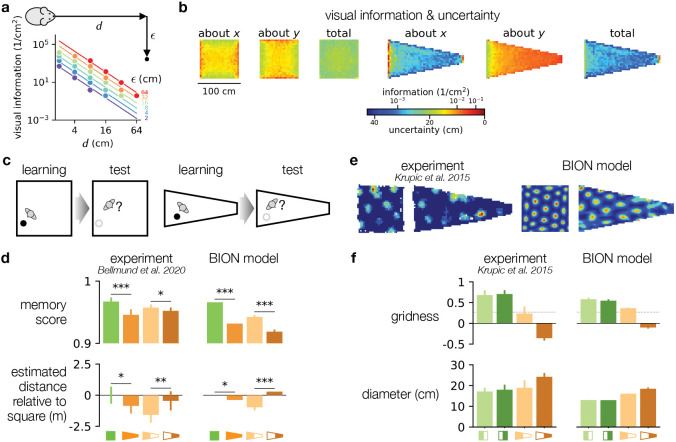
Deformations arise from inhomogeneous uncertainty. **a.** *Inset*: top-down view of an observer (rodent in this case) and a point landmark (solid black circle), at a given distance along the line of sight (*d*) and ‘eccentricty’ (ϵ, distance orthogonal to the line of sight) from the observer. *Main plot*: ideal observer’s visual (Fisher) information about *d* as a function of the true distance (abscissa) and eccentricity of the landmark (colors), lines show analytic approximation, markers show numerical simulation. Note logarithmic scale for both axes as well as color scale. **b.** Visual information about x- and y-coordinates of location, and total information in the square and trapezoid environments. Warmer/cooler color indicates more/less information, and conversely, lower/higher uncertainty. **c.** Homing task. Participants memorized the location of an object during the learning phase (filled circle), to which they were asked to return (after the object was hidden, open circle) during the test phase. Two kinds of environments were used: square (left) and trapezoid (right). **d.** Homing performance from experimental data (left^5^) and BION model predictions (right). Each set of four columns shows square vs. trapezoid environments and broad vs. narrow part of the trapezoid environment (see filled areas in schematics at the bottom). *Top row*: memory score (rank of homing location’s distance to target compared to all possible locations). *Bottom row*: estimated distance between target locations in each (half) of the environment compared to that in the square (true distances were matched). Error bars show s.e.m. across subjects. **e-f.** Example grid fields (**e**) and grid field characteristics (**f**) in experimental data (left^6^) and BION model predictions (right). **e.** Grid fields of the same cell in a square and a trapezoid environment. **f.** Gridness (top) and diameter of grid fields (bottom). Each set of four columns shows data for grid fields in the left and right halves of the square and trapezoid environments separately (see filled areas in schematics at the bottom). Error bars show s.e.m. across neurons.

A critical insight of the BION model is that the same landmark provides different amounts of visual information about the subject’s location, depending on its distance along, *d*, and eccentricity from, ϵ, the subject’s line of sight (Fig. 2a, inset). For example, the landmark could be a corner of the enclosure, with *d* being the subject’s distance from the corresponding wall when facing it. In particular, based on the basic laws of optics describing a pinhole projection with finite resolution, we were able to show analytically that the visual information (*I*, formally the Fisher information) about the distance (*d*) scales with the square of eccentricity (ϵ) and inversely with the fourth power of the distance itself (*d*):

(1) $$I\propto \frac{\epsilon^{2}}{d^{4}}$$

Importantly, we were also able to show that the same expression holds for a more realistic scenario, with a fully rendered retinal image and a finite-sized retinotopic neural population whose signaling is corrupted by Poisson noise (Supp. Material). Indeed, numerical simulations of the visual likelihood of current location using the BION model confirmed these calculations (Fig. 2a).

Eq. 1 makes direct predictions about how visual information about location changes inside a square- versus a trapezoid-shaped environment, such as those used in several experiments^5,6^ (Fig. 2b). In general, because information falls steeply with *d*, overall information at any location is dominated by information obtained while looking in the direction of the closest wall. Thus, in both kinds of environments, information is higher near the walls. This is consistent with humans making smaller homing errors near walls^5^. More importantly, due to the fundamental symmetry of a square environment, information has a symmetrical distribution, and is also symmetric with respect to the axis along which spatial position needs to be determined (x or y in Fig. 2b). In contrast, in the trapezoid, information is asymmetric between its two sides, and (again consistent with empirical data^5^) overall smaller about position along the long (x-) axis (because distances are greater) than about position along its short (y-) axis (Fig. 2b).

In homing tasks (Fig. 2c, cf. Fig. 1c), total errors are dominated by the more uncertain direction, i.e. position about the long axis in the trapezoid (Fig. 2b, compare left two panels for the trapezoid, showing uncertainty about x and y separately, with the last panel, showing total uncertainty). Along this direction, on average, *d* is greater and thus information is smaller than along either direction in the square. Therefore, the BION model predicts homing performance to be worse in the trapezoid than in the square, which is what has been found experimentally^5^ (Fig. 2d, top, green vs. orange). Finally, the asymmetry between the two ends of the trapezoid results in ϵ being larger and thus information being greater near the broad end. As a consequence, homing performance is predicted to be relatively better near its broad end than near the narrow end, again consistent with data^5^ (Fig. 2d, top, light vs. dark brown).

Note that path integration alone cannot explain these results, as it predicts an increase in uncertainty with time, not as a function of space. This may contribute to spatial information decreasing with *d* (and therefore the decrease of information away from the walls, and the overall increased uncertainty in the trapezoid than in the square environment that we described above) as long as there is an additional landmark-based mechanism that resets path integration near the walls. However, even such a hard reset mechanism cannot account for the scaling of information with ϵ that underlies the BION model’s predictions about differences between the two halves of the trapezoid.

In another task used in the same experiment, participants had to estimate distances between objects. Overall, participants showed different degrees of estimation biases depending on the geometry of the environment (Fig. 2d, bottom left). Interestingly, however, the pattern of these estimation biases did not simply reflect the patterns seen in memory performance: while the biases in the trapezoid were overall more negative than in the square (in line with memory performance being poorer in the trapezoid than in the square), they were *less* negative (although memory performance was *more* diminished) in the narrow side of the trapezoid than in the broad side (Fig. 2d, left, compare bottom to top).

The ideal observer reproduced this pattern of results (Fig. 2d, bottom right). The relative underestimation in the trapezoid was due to regression to the mean: as visual information is always uncertain, it needs to be combined with the prior covering the extent of the environment. This biases locations to be estimated as being closer to the middle of the environment (the mean of the prior) and thus overall distances between them to be smaller – and more so in the trapezoid because visual information is more uncertain there overall (Fig. 2b). The more subtle differences in underestimation between the two halves of the trapezoid environment were explained by the interplay of how environmental geometry changes both visual information (Eq. 1, i.e. the likelihood) and the prior (Supp. Material).

In the BION model, predicted grid field sizes depended on the overall level of spatial information, and their irregularities depended on the degree of warping that was necessary to achieve the level of spatial anisotropy in the uncertainties expressed by the ideal observer (Fig. 1d, and Fig. 2e, right). Compared to the square enclosure, uncertainty in the trapezoid was greater and more anisotropic (differing greatly along the two axes of the enclosure, Fig. 2b). Thus, the BION model predicted larger grid fields with lower levels of gridness for the trapezoid than for the square (Fig. 2f, right). For analogous reasons, the model also predicted larger grid fields with lower levels of gridness for the narrow than for the broad side of the trapezoid (Fig. 2f, right, orange). These predictions matched experimental measurements of grid fields in such square and trapezoid environments^6^ (Fig. 2e, left, f, left).

### Scaling arises from model mismatch

**Fig. 3 |:**
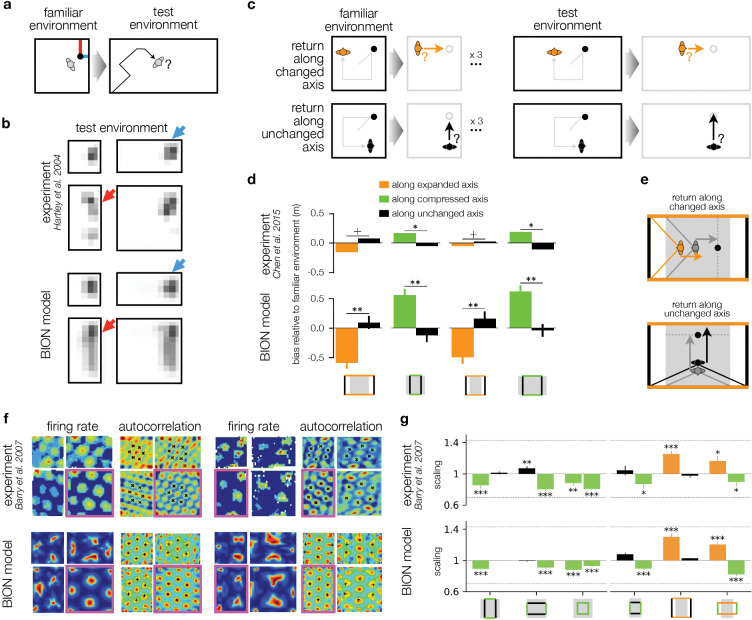
Scaling arises from model mismatch. **a.** Homing in scaled environments^8^. Human participants first learned a target location (black circle) in a familiar environment (e.g. a square room) and were asked to return to that location (black path) with the target now invisible in a test environment that was either the same as the familiar environment or, unbeknownst to them, scaled (e.g. a rectangular room as shown). Blue and red lines show distances to the nearest walls along the horizontal and vertical axes, respectively. **b.** Homing location distributions for the square familiar environment and target object location shown in **a** in experimental data (top^8^) and the BION model (bottom). Each sub-panel shows a different test environment. Blue/red arrows show conditions where the the target object’s distance to the nearest wall along the axis of scaling was comparatively smaller/larger (see blue/red lines in **a**). **c.** Axis-dependent homing in scaled environments^9^. Participants first walked from the center of an environment to a visible target and were asked to remember its location (top row, black circle). The target then disappeared and participants were guided across the environment along a random, multistep path (gray ‘outbound’ path). Participants were then asked to return to the location of the target (orange and black arrows) in the absence of any visual input (gray bounding box). In each block, three trials were performed in a ‘familiar’ environment (left) and then a single trial was performed in a scaled test environment (right). The beginning of the return path (i.e. the endpoint of the outbound path) was always such that the direction to the target was either along the axis that was changed in that trial (top, orange arrows), or along the unchanged axis (bottom, black arrows). **d.** Homing biases in test environments along the changed (orange and green bars) and unchanged (black bars) axes observed in the data (top^9^) and the BION model (bottom). Schematics in the bottom show familiar environment (gray) and test environment boundaries (black and colored lines) for reference. Error bars show s.e.m. across simulated subjects (not drawn for the experimental data because only the significance of the comparison and mean differences were available). **e.** Schematic of how biases arise when the test environment is scaled (orange) from the familiar environment (gray). When the return direction is along the expanded axis (top), the angle subtended by the corners of the far wall (orange), as seen during the outbound journey in the test environment, matches the angle at a location in the familiar environment (gray) that is closer to the target location (black circle). This results in an undershoot during the return journey (negative bias, orange vs. gray arrows). Conversely, for homing along the unchanged axis (bottom), the angle subtended by the corners of the far wall in the test environment (black) suggests being farther from the target in the familiar environment (gray), hence leading to an overshoot (positive bias, black vs. gray arrows). **f.** Effect of environmental scaling on rodent grid cell responses^10^. Rats were first familiarized with one room (pink outline, square and rectangular room in left and right panels, respectively) and then were introduced to scaled rooms (three adjacent rectangles). Grid cell firing rates are shown on the left and autocorrelations on the right for each room shape. **g.** Grid field scaling ratio along the changed axis (green and orange for compressed and expanded, respectively) and along the unchanged axis (black) in the data (top)^10^ and the BION model (bottom). Schematics in the bottom show familiar environment (gray) and test environment boundaries (black and colored lines) for reference. Error bars show s.e.m. across neurons.

The BION model also explains scaling of behavioral and neural response patterns in unfamiliar environments as a consequence of the mismatch between the internal model and the sensory input. In a typical human behavioral experiment^8^, participants were familiarized with a particular environment and their homing to a previously experienced target location was tested, after the environment had been expanded or compressed along one or both axes unbeknownst to them (Fig. 3a shows expansion along horizontal axis). Because the changing of the environment in the test phase was unknown to participants, we modeled the ideal observer’s inferences as being computed in the familiar environment’s frame of reference, resulting in a model mismatch between the internal model used by the ideal observer and the actual environment (Methods). Due to this model mismatch, the actual sensory inputs (that were generated by the test environment) led to higher uncertainty in the ideal observer (that interpreted them according to the familiar environment), particularly along the axis of the environment that was changed, and particularly for locations along the axis for which information was low to begin with (because they were farther from the nearest wall, see above). Thus, in line with experimental data^8^ (Fig. 3b, top), the BION model (Fig. 3b, bottom) predicts response variability to be higher along the changed axis especially when the distance to the closest wall along that axis is greater (Fig. 3b, red vs. blue).

Unfamiliar environments do not only lead to increased response variability, but also to systematic biases in homing responses. These biases have been characterized in an experiment in which participants were required to return to a previously visited target without visual input after they were led away from it (in the presence of visual input) along an experimentally controlled path (Fig. 3c, gray trajectories in left panels followed by orange or black arrows in right panels). Critically, participants performed this task for three consecutive trials with a fixed (‘familiar’) environmental geometry, and then on the fourth trial they performed it either in the same environment, or in an unfamiliar ‘test’ environment that was scaled (expanded or compressed) along one of its axes (Fig. 3c shows the case when the test environment is expanded along the x-axis). Participants were specifically led to believe that the test environment was the same as the familiar one although it was in fact scaled – corresponding to a model mismatch. Response biases (the length of the return journey in the scaled environment relative to that in the familiar environment with a matched distance to the target) were measured separately for return journeys along the changed (Fig. 3c, top, orange) or unchanged axis of the environment (Fig. 3c, bottom, black arrow).

Responses were negatively biased (i.e. undershot) along an expanded axis (Fig. 3d, top, orange bars) and positively biased (i.e. overshot) along a compressed axis (Fig. 3d, top, green bars). The BION model captured this because, during the outward journey, the visual image of the far wall along the expanded axis suggested that this wall was still at a great distance, which in turn made the outward journey seem shorter than it actually was, leading to an undershoot in simulated responses (Fig. 3d, bottom, orange and Fig. 3e, top) – and vice versa when the far wall was seen along the compressed axis (Fig. 3d, bottom, green). Interestingly, even responses along the unchanged axes were biased, and in the opposite direction: positively (overshooting), when the environment was expanded, and negatively (undershooting), when it was compressed (Fig. 3d, top, black bars). The BION model also captured this behavior simply because looking along the unchanged direction showed the farther expanded wall (that was seen more during the outward journey) subtend a large angle, making it appear as if it was closer, and thus the outward journey longer than it actually was, leading to an overshoot in simulated responses (Fig. 3e, bottom) – and again vice versa in the compressed environment (Fig. 3d, bottom, black bars).

Although the geometrical intuitions above only apply to the effect of visual inputs, and are most straightforward assuming simple point-like landmarks (the corners of the enclosure), the simulation results we show were obtained by the full ideal observer that uses a rendered retinal image as visual input, and combines it with self-motion inputs (Fig. 1a–b). This shows that the basic laws of optics determining the retinal projection can have significant behavioral effects in a Bayes-optimal observer during navigation – and thus explain the experimentally observed pattern of results.

Recordings of rodent grid cell responses in analogous scaled rectangular enclosures found that the regularity (specifically, isotropy) of grid fields depended on whether a given enclosure shape was familiar (pink outlined environments in Fig. 3f) or encountered after scaling, rather than on the shape itself. For example, grid fields in the identical square environment were isotropic when the square was familiar (Fig. 3f, top left, firing rate and autocorrelation map inside pink outline), but became more anistropic when the familiar environment was a rectangle and the square environment was encountered as a scaled version of this rectangle (Fig. 3f, top right). Conversely, grid fields were isotropic in the rectangular environment in the second case but anisotropic in the first case. These results were naturally reproduced by the BION model, simply because the distortions of uncertainty due to model mismatch, which explained human behavior, required increased warping of grid fields (Fig. 3f, bottom).

In the same set of experiments, a finer grained analysis revealed systematic distortions of grid fields along the changed vs. unchanged axes of these environments (Fig. 3g, top). In particular, in line with the biases seen in human behavior (Fig. 3d, top), grid fields compressed and expanded respectively along the compressed and expanded axes of the environment (Fig. 3g, top, green and orange bars), and showed opposite scaling along the unchanged direction (Fig. 3g, top, black bars). Once again, the same distortions in the BION model’s spatial uncertainty that explained human behavior, also required grid field warpings that reproduced these neural response patterns (Fig. 3g, bottom).

### Tethering arises from cue conflict

**Fig. 4 |:**
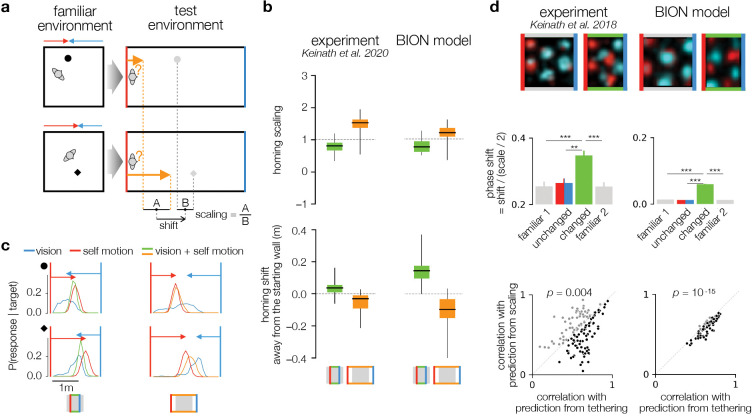
Tethering arises from cue conflict. **a.** Distance-dependent homing in scaled environments^12^. *Left.* Participants learned locations of four nameable objects (only two shown here for simplicity, black circle and diamond) in a square familiar environment. The objects were at different distances from the walls (red and blue arrows indicate the distance from two opposite walls along the axis that is to be changed). *Right.* In a test trial, participants were asked to return to the location of an object cued by its name in an expanded (as shown) or compressed test environment, starting from one of the four walls (for this trial, the starting wall is red and the opposite wall is blue for illustration purposes only). Gray circles show the ‘equivalent’ location of each target object (hidden from participants), i.e. the location that is at the same distance from the center of the enclosure in the test as in the familiar environment (note that this preserves distances between objects). Schematics in the bottom give intuitive explanation for main experimental measures (actual calculations used linear regression across 4 objects × 4 starting walls with the predicted shift appropriately changed depending on the starting wall^12^). The scaling of homing responses measures the ratio of the distance (A) between the two homing locations (orange) and the distance (B) between the target locations (or equivalent target locations, gray). The shift of responses measures the difference between the average of the homing locations (orange arrows) and the average of equivalent target locations (gray), with a sign that is defined to be positive when the shift is away from the starting wall. **b.** Distribution of scaling and shift in the data (left; Fig. 6d in Ref. 12, with an opposite definition of the sign of shift as we use here) and the BION model (right) is consistent with tethering. Box-and-whisker plots show range (whiskers), interquartile range (box), and median (horizontal line) across subjects. **c.** Distribution of homing locations simulated by the BION model, when the test environment is compressed (left column) and expanded (right column), for a target nearer the starting wall (top row) and farther from the starting wall (bottom row). Red and blue vertical lines indicate the starting and opposite walls, corresponding arrows show the distance of the object to these walls in the familiar environment shown in **a**. Curves of different colors indicate the distribution of homing responses using both visual and self motion (green and orange), vision alone (blue, mostly preserving the original distance to the blue wall), and self motion alone (red, mostly preserving the original distance to the red wall). Schematics in the bottom show familiar environment (gray) and test environment boundaries (colored lines) for reference. **d.** *Top row.* Grid fields in the data (left^11^) and the BION model (right) show tethering analogous to that seen in behavior. The familiar, training environment is a square and the test environment is compressed along the horizontal axis. Red and cyan shading show firing rates conditioned on the last approached wall being the west and east wall, respectively (walls are colored in the figure for visualization only). Note that in the square environment, the cyan shading overlaps the red shading because the phases of the grid fields are the same regardless of the last wall approached. *Middle row.* Phase shift of the grid fields relative to the scale of the grid (distance between adjacent peaks) in the familiar environment (gray, before [1] and after [2] experiencing the novel environment) and along the unchanged (red/blue) and changed (green) axes of the compressed environment. Note that, due to the periodicity of grid fields, their shifts can only be measured up to half the distance between them, i.e. half their scale. Error bars show s.e.m. across neurons. *Bottom row.* Pearson’s correlation between the observed tuning curves conditioned on the last wall being on the changed axis and the tuning curves predicted from perfect scaling (ordinate) vs. perfect shifting (i.e. boundary tethering, abscissa). Each point is a neuron, colored black if tethering correlation is bigger than scaling correlation, and gray otherwise. P-values show the significance of a paired t-test in favor of tethering vs. scaling across neurons (comparing Fisher-transformed correlations).

Unexpected changes in environmental geometry have also revealed an intriguing ‘tethering’ effect in both behavioral and neural responses. In behavioral experiments, homing end point locations corresponding to different targets in the changed geometry have been compared to the original locations of those targets in the familiar geometry (Fig. 4a). The relationship between these two sets of target locations was characterized by a ‘scale’ factor, measuring the ratio of distances between homing locations and distances between original target locations, and an overall ‘shift’ in homing locations away from the wall from which participants started their return journeys (‘starting wall’) in a given trial with the changed geometry. As expected, the scaling of homing locations reflected the scaling of the environment: when the environment was compressed or expanded, homing locations were respectively compressed or expanded (Fig. 4b, top left). However, there was also a systematic shift in homing locations (Fig. 4b, bottom left): when the environment was compressed, they shifted forwards, while in expanded environments, they shifted backwards, keeping their distance to the starting wall similar to that in the familiar environment in both cases (i.e. as if they were ‘tetherted’ to the starting wall)^12^.

The BION model readily explained these results as a fundamental feature of navigation in the face of spatial uncertainty. According to the model, interpreting visual and self-motion inputs in the changed geometry under the internal model of the familiar geometry leads to cue conflict between these two inputs. That is, when path integration using self-motion inputs on a return journey suggests that some distance has been traveled since leaving the starting wall, visual inputs are in conflict with this, because the opposite wall appears nearer or farther depending on whether the environment has been compressed or expanded. In other words, path integration anchors responses to the starting wall, while visual inputs tend to anchor them to the opposite wall (Fig. 4c, red and blue distributions peaking at the ends of corresponding arrows). Critically, the ideal observer combines these two cues according to their respective uncertainties, such that the final estimated spatial location depends more on the input with less uncertainty. In general, uncertainty from path integration grows with distance traveled from the starting wall (Fig. 1b), while uncertainty in visual inputs decreases because of its inverse scaling with the distance from the opposite wall (Eq. 1). Indeed, when we simulated the ideal observer with each input separately, response distributions based on only self-motion input became slightly broader for targets farther from the starting wall (Fig. 4c, red distributions in bottom compared to top). In contrast, response distributions based on only visual inputs were substantially broader for targets nearer the starting wall compared to more distant targets (Fig. 4c, blue distributions in top compared to bottom), as nearer targets are farther from the opposite wall. Therefore, responses to targets near the starting wall were dominated by path integration, and were thus mostly tethered to (i.e. kept their distance from) the starting wall (Fig. 4c, top row, green and orange response distributions largely overlap with red distributions). In comparison, homing to targets near the opposite wall relied more on visual inputs, whose reliability was higher there, and thus these responses were tethered relatively more to the opposite wall (Fig. 4c, bottom row, green and orange response distributions interpolate between red and blue distributions).

The opposite tethering of near and far targets to the starting and opposite walls in the BION model, respectively, explains the experimentally observed patterns of scaling of response distributions in compressed and expanded environments (Fig. 4b, top right). The model also predicted the experimentally observed overall tethering of responses to the starting wall (Fig. 4b, bottom right) because, compared to other experiments, self-motion inputs were more informative than visual inputs in this experiment, as subjects walked a short, straight path from a known location (center of an identifiable wall). This contrasts with some of the other experiments we modeled above, in which visual inputs were relatively more informative because subjects started from a random location uniformly sampled within the environment (Fig. 2c) or walked a long meandering path before the homing phase (Fig. 3c).

A re-analysis of previous experimental data^10,42^ revealed that, similar to human homing responses, rodent grid fields are also tethered to environmental boundaries under analogous conditions (i.e. unexpected environmental scaling). In particular, the distance of a given grid field to the wall that the animal most recently approached (i.e. was within 12 cm of it) is preserved between familiar and compressed test environments^11^ (Fig. 4d, top left). In order for the neural posterior to follow the overall tethering of the ideal observer’s posterior to the last wall approached, which was apparent in the behavioral data (Fig. 4b, right), the BION model also predicted this tethering of neural tuning curves (Fig. 4d, right; Supp. Material). Further quantifying tethering as a relative phase shift of grid fields confirmed that it was significantly larger along the changed axis than along the unchanged axis of the compressed environment, and also larger than in the familiar environment before and after testing in the compressed environment both in experiments and in the model (Fig. 4d, middle left and right). Finally, empirical changes in tuning curves in the compressed environment were also compared to hypothetical deformations based on pure scaling vs. pure tethering. Although, overall, these two kinds of deformations were found to provide comparable fits to the empirical changes, tuning curves were significantly better correlated with tethering-based predictions in experiments as well as in the model (Fig. 4d, bottom left and right).

## Discussion

Our work provides a unifying, normative theory of how both behavioral and neural response patterns depend on environmental geometry. The key insight is that, due to the basic laws of optics, visual inputs have a fundamental effect on spatial uncertainty, which in turn is reflected in behavioral responses and encoded in neural responses as deformation, scaling, and tethering by environmental boundaries. We quantify these effects through numerical simulations of the BION model, and provide mathematical insights into their main qualitative features by analytical derivations.

Previous models that attempted to link behavior or grid cell properties to environmental geometry only explained a subset of the phenomena that we predict, and typically accounted for behavioral effects as mere epiphenomena of particular neural representations that were themselves often posited to be based on idiosyncratic features of the environment^8,9,11,12^. In particular, based on observations of place cell responses, the ‘boundary vector hypothesis’ postulated a special status for environmental boundaries, and their representation by specialized cell types^7^ – a prediction confirmed by later experiments^43–45^. These cells were in turn suggested to underlie some of the behavioral^8^ and neural^11,46^ effects seen during environmental deformations. In contrast, the BION model does not rely on explicit notions of walls or boundaries, or externally provided identifying tags for them. Instead, it provides a normative basis for the special status of environmental boundaries in navigation by only using ‘raw’ visual inputs. Thus, it explains the behavioral and neural effects of boundaries from first principles, as consequences of the higher visual information close to such boundaries (Fig. 2a, Eq. 1). In line with this, the dependence of hippocampal place cell responses on environmental boundaries has recently also been hypothesized to reflect such information-theoretic principles^47^. Taken together, these results raise the possibility that the very existence of boundary vector cells^43^ (and related boundary^44^ and border cells^45^) may also derive from the same normative principle.

The BION model starts from the fundamental principle (long-recognized in engineering applications) that representing spatial uncertainty is key for efficient and reliable navigation because allocentric location can only be inferred from sensory inputs and motor outputs that are inherently noisy and ambiguous. This is a fundamental difference from dominant theories of grid cell responses that either make the simplifying assumption that location is already given to the model as an input, without uncertainty^13,17,38,46,48,49^, or perform localization from sensory inputs without considering the unavoidable uncertainty in the process^39,50^. Conversely, models that formalized navigation in a Bayesian inference framework have not incorporated an image-computable ideal observer. Thus, these models did not study how environmental geometry affects spatial uncertainty and, in turn, homing behavior or grid cell responses^16,20,21,25,51–56^.

An alternative theoretical account of some forms of grid field deformations is based on the successor representation (SR)^57^. Specifically, according to SR-theory, place cell responses encode a predictive map, the (time-discounted) probability with which a given location in the environment will be reached from the animal’s current location, while grid fields provide a set of basis functions for efficiently representing this map^13^. Because these transition probabilities depend on the geometry of the environment, they inherit the asymmetry of the environment. Therefore, SR predicts an overall asymmetry of grid fields between the two halves of a trapezoid environment^13^, and lower gridness in the narrow side^46^. Based on visual uncertainty alone (Fig. 2b), the BION model also predicts this overall asymmetry, along with more fine-grained differences in the gridness and diameter of grid fields (Fig. 2f). While SR-based theories have also made predictions about grid field diameters (or equivalently, radial frequencies, Fig. 1j in Ref. 5), these seem to be in conflict with experimental data that shows larger diameters in the narrow than in the broad half of the trapezoid^6^ that the BION model successfully predicted (Fig. 2f). In addition, unlike the BION model, SR does not explain experimental findings when the environment is changed (Figs. 3 and 4).

The recently proposed ‘default representation’ (DR) is similar to SR, in that it also encodes a predictive map without considering spatial uncertainty^49^. DR mainly differs from SR in that it is based on a ‘default’ behavioral policy that depends on basic physical constraints of the environment rather than the current task-dependent behavioral policy. Therefore, although the predictions of DR have not been spelled out for the experiments we considered here, they can be expected to be similar to those of SR, as long as the behavior of the animal is constrained mainly by environmental geometry (as in random pellet chasing, but see below).

Critically, SR-like and our uncertainty-based mechanisms need not be mutually exclusive. For example, there are changes in place cell responses (albeit not in grid cell responses^58–60^) that happen in the absence of any change in visual inputs, when only the behavioral policy of the animal is changed^61^. Such changes may be naturally accounted for by SR, as changing the behavioral policy will also change the transition probabilities between locations, but might not be readily predicted by our uncertainty-based account. Conversely, the grid field deformations described for unexpected environmental changes that the BION model naturally captured (Figs. 3 and 4) seem difficult to reconcile with a purely SR-based account. Moreover, studies of grid field deformations using a ‘hairpin maze’ have found profound differences in grid cell responses when subjects navigated the same environment with vs. without walls, even though their trajectories (and hence transition probabilities) were constrained to be similar^58,60^. These results challenge a purely SR-based (but not DR-based) account of grid cell firing^49^, while they are consistent with an uncertainty-based mechanism given the critical role of visual inputs in determining spatial uncertainty, as revealed by our analyses. Taken together, we propose that SR (or DR) may be most relevant for place cell responses, while grid cell deformations may be driven primarily by uncertainty-based mechanisms.

Our work focuses on how a probabilistic estimate of location (and head direction) is computed from noisy and ambiguous sensory inputs. A complete understanding of navigational behavior also requires the formalization of how these estimates, and the uncertainty about them, are used for route planning^62^ – something that we only modeled phenomenologically (Fig. 1c). Indeed, recent work using a more formal model of the planning process^20^ has been able to account for apparent violations of the Bayes-optimal combination of landmark and self motion cues^27,28^ and reconcile them with earlier results suggesting optimal cue combination^26^. These results lend further support to our core suggestion: that Bayesian principles play a fundamental role in navigation.

## Methods

### Choice of experimental tasks

We chose experimental tasks with which we can test both behavioral and neural effects of uncertainty as a function of the environmental geometry. Typically, human experiments yielded suitable behavioral measures (e.g., endpoints in homing tasks^5^) and rodent experiments yielded suitable neural measures (e.g., electrophysiological recordings from grid cells while the animal chased a randomly dropped pellets). Therefore, we chose pairs of experiments that employed analogous environmental geometry and compared their results with our model (Table 1).

### BION ideal observer model

#### Generative model

We consider an observer (a modeled human or nonhuman subject) that navigates an environment while trying to find out its own location within the environment. In experiments we modeled, the subjects were familiarized with the environment, or believed that they were in a familiar environment, so we assume that the observer’s uncertainty about the configuration of the environment (also known as a ‘map’) to be negligible. The observer still has uncertainty about where it is, due to noise in its sensory inputs (visual, self-motion, and tactile) and its movements.

Formally, at each time step *t*, the observer intends to turn by *u*^*θ*^, which results in a noisy rotation ${\tilde{u}}^{\theta }$ in its allocentric head direction *θ*:

(2) $${\tilde{u}}_{t}^{\theta }\sim \mathrm{vM}\left(u_{t}^{\theta }\Delta t,\;\kappa \Delta t\right)$$

(3) $$\theta_{t}=\theta_{t-1}+{\tilde{u}}_{t}^{\theta }$$

where vM(·) denotes von Mises distribution. Likewise, the observer intends to move forward by *u*^x^, which results in a noisy forward motion ${\tilde{u}}^{x}$ in its allocentric location *ℓ*, except when it meets a wall ∂ℰ, in which case it stops at the nearest intersection of its trajectory and the wall:

(4) $${\tilde{u}}_{t}^{\text{x}}\sim \mathrm{gamma}\left(u_{t}^{\text{x}}\Delta t,\;{\left(\sigma^{\text{x}}\right)}^{2}u_{t}^{\text{x}}\Delta t\right)$$

(5) $${\tilde{u}}^{\ell }:={\left({\tilde{u}}^{\text{x}},\;0\right)}^{⊤}$$

(6) $$\ell_{t}^{\prime }=\ell_{t-1}+R\left(\theta_{t}\right){\tilde{u}}_{t}^{\ell }$$

(7) $$ℬ\left(\ell ,\;\ell^{\prime }\right):=\bar{\ell \ell^{\prime }}\cap \partial ℰ$$

(8) $$\ell_{t}=\{\begin{array}{ll} \ell_{t}^{\prime } & \;\text{if}\;\ell_{t}^{\prime }\in ℰ\\ {\mathrm{argmin}}_{\ell \in ℬ\left(\ell_{t-1},\ell_{t}^{\prime }\right)}d\left(\ell_{t-1},\;\ell \right) & \;\text{if}\;\ell_{t}^{\prime }\notin ℰ \end{array}$$

The observer receives egocentric visual input on a flat rectangular array of retinotopic neurons through a pinhole projection of the 3D scene. The individual neuron’s signal *V* is subject to Poisson noise with the tuning curve (i.e., expected firing rate) given by a blurred first person view $\bar{v}$, which is a function of the observer’s location and head direction:

(9) $$V_{t}^{i}\sim \mathrm{Poisson}\left({\bar{v}}_{i}\left(\ell_{t},\;\theta_{t}\right)\Delta t\right)$$

Finally, the observer receives egocentric tactile input *W* if it is within distance *x*_w_ from a wall, with reliability *p*_w_:

(10) $$W_{\gamma }\sim \{\begin{array}{ll} \text{Bernoulli}\;\left(\frac{1}{2}+\frac{1}{2}p_{\text{w}}\right) & \;\text{if}\;\left|ℬ\left(\ell ,\;\ell +x_{\text{w}}u(\theta +\gamma )\right)\right|>0\\ \text{Bernoulli}\;\left(\frac{1}{2}-\frac{1}{2}p_{\text{w}}\right) & \text{otherwise} \end{array}$$

There were a few differences between the generative models we used for different experiments: (1) For human experiments, which were performed in VR, we did not use tactile input (Eq. 10). (2) For the scaling experiments done in large environments^8,9^, we used isotropic Gaussian distribution instead of gamma distribution for the translation (Eq. 4).

A recent theory used a normative model to explain undershoot in navigation to a remembered target location by having a prior that places higher probability on slower speeds (i.e. a slowness prior rather than imperfect integration)^22^. In contrast, in our model we assumed that the perception of the control signal is well calibrated and is observed, while the resulting movement is noisy, following the convention of some of the robotic navigation literature^19^. That said, as our model specifies P(Δ(*ℓ*, *θ*)|(**u**^*ℓ*^, *u*^*θ*^)), it would be possible to emulate a slowness prior. However, we did not include slowness prior in our analyses because (1) in the experiments we modeled, vestibular and/or afferent copy of motor signals (in addition to optic flow) were available, which would have made the perception of self motion more reliable, compared to the experiment modeled by Ref. 22 where only optic flow was available; (2) the main results we replicated were differences between conditions (e.g., difference of biases between expansion vs. compression of environments) rather than the bias relative to the true distance, so that any undershoot would be subtracted in the comparison.

#### Bayesian filtering

The ideal observer is given by Bayesian inversion of the generative model above. Specifically, we used Bayesian filtering, where the observer infers the posterior distribution over its allocentric pose (location and head direction) given the full history of noisy sensory inputs and control signal.

(11) $$\text{P}\left(z_{T}=z|u_{0:T},\;o_{0:T}\right)\propto \int \underset{\text{observation}}{\underset{︸}{\text{P}\left(o_{T}|z_{T}=z\right)}}\underset{\text{prediction}}{\underset{︸}{\text{P}\left(z_{T}=z|u_{T},\;z_{T-1}=z^{\prime }\right)}}\underset{\text{dynamic}\;\text{prior}}{\underset{︸}{\text{P}\left(z_{T-1}=z^{\prime }|u_{0:T-1},\;o_{0:T-1}\right)}}\;dz^{\prime }$$

where the pose is the latent state **z** := (*ℓ*, *θ*) that consists of the allocentric location *ℓ* and head direction *θ*, and observation **o** := (**v**, **w**) consists of egocentric visual input **v** and tactile input **w**, which we assume to be independent given **z**. Hence, P(**o**|**z**) = P(**v**|**z**) P(**w**|**z**). For simplicity, we assume that the control signal **u** (= intended movement) is known by the observer without noise, but that the actual movement is noisy^19^.

### Visual likelihood

A key novel component of the BION model is its visual likelihood. It is computed as the likelihood of the egocentric visual observation given an allocentric pose. This is achieved by inverting the generative model of the visual input (Eq. 9). Specifically:

(12) $$\text{P}(V=v|z)=\prod_{i}\frac{{\left({\bar{v}}^{i}(z)\Delta t\right)}^{r^{i}\Delta t}}{\left(r^{i}\Delta t\right)!}e^{-{\bar{v}}^{i}(\text{z})\Delta t}$$

where *i* indexes neurons with a retinotopic arrangement (‘retinotopic neural population’) and **z** := (*ℓ*_*t*_, *θ*_*t*_). This is an extension of a classical Bayesian decoder (Eq. 3.30 of Ref. 32) to the 3D case, by using tuning curves obtained from the projection of a 3D scene onto the retinal plane, ${\bar{v}}^{i}(z)$. This extension has an important implication for the amount of visual information. While the assumption of independent Poisson noise of pixels is an obvious simplification, it still yields powerful predictions when combined with the law of optics of the projection from the 3D world onto the 2D retina (Supp. Material and Results). The extension also has implications for numerical computation. To see this, note that expressions like the above is typically evaluated using logarithm for numerical stability as:

(13) $$\log\text{P}(V=v|z)=\sum_{i}\left[r^{i}\log{\bar{v}}^{i}(z)-{\bar{v}}^{i}(z)\cdots \right]$$

Here, unlike Eq. 3.31 of Ref. 32, we cannot ignore the second term, because we cannot assume it will be independent of **z**. That is because in case of Ref. 32, the tuning curves are arranged evenly in the space of the stimulus, whereas in our case, the space of the stimulus, **z**, cannot be linearly mapped onto the retinal space (on which the tuning curves are approximately evenly arranged). As a result, for example, some places **z** may simply be darker than other places and evoke fewer spikes across the retinal space, making the second term different between different **z**.

### BION neural representation model

We use the readout *P*^g^(*ℓ*) of a Bayesian decoder of the animal’s location *ℓ* given Poissonian firing rates **r** of a grid cell population as ‘neural posterior’. Our hypothesis is that the tuning curves of the grid cells are warped such that this neural posterior represents the ideal observer’s posterior P^b^ as closely as possible. Formally, the neural posterior has a parametric form obtained by inverting the generative model of the firing rates:

(14) $$r_{i}\sim \mathrm{Poisson}\left(f_{i}^{{ℰ}^{*}}(z)T\right)$$

(15) $$P^{\text{g}}\left(\ell ;\;r,\;\Theta^{\text{w}}\right)\propto \text{P}{\left(\ell |r;\;f^{\tilde{ℰ}}(z)\right)}^{\alpha^{\prime }}$$

(16) $$\;\propto {\left[\text{P}\left(r|\ell ;\;f^{\tilde{ℰ}}(z)\right)\underset{⊥\ell }{\underset{︸}{\text{P}\left(r;\;f^{\tilde{ℰ}}(z)\right)}}\right]}^{\alpha^{\prime }}$$

(17) $$\log P^{\text{g}}\left(\ell ;\;r,\;\Theta^{\text{w}}\right)=\alpha^{\prime }\sum_{i}\log\text{P}\left(r_{i}|\ell ;\;f_{i}^{\tilde{ℰ}}(z)\right)+(\text{const)}$$

where *i* indexes neurons, $f^{{ℰ}^{*}}$ is the ‘encoding’ tuning curve, which gives rise to the firing rate **r** := {*r*_*i*_}, and $f^{\tilde{ℰ}}$ is the ‘decoding’ tuning curve. Here $\tilde{ℰ}$ is the familiar environment, and ${ℰ}^{*}$ is the test environment, which may or may not be the same as $\tilde{ℰ}$. *α′* is a parameter that determines the sharpness of the posterior, which ensures that the neural posterior does not become infinitely sharp even with infinite number of neurons. It is necessary because we assume that there is nonzero uncertainty in the ideal observer’s posterior due to noisy observation (see Results).

Our strategy is to first optimize the tuning curves to match the neural posterior with the ideal observer’s posterior, and then to compare them with the experimental data. For this comparison, for the neural model, we chose, **z** := (*ℓ*, *h*) where *ℓ* is the location of the animal and *h* is the last wall approached, to match the way the tuning curves are reported in Ref. 11 (Fig. 4d). Specifically, we fit $f^{{ℰ}^{*}}$ (observable), parameterized with **Θ**^w^ to be a warping with a mesh. When the test environment is familiar, $f^{\tilde{ℰ}}$ is constrained to be the same as $f^{{ℰ}^{*}}$, which is fit. Otherwise, $f^{\tilde{ℰ}}$ is fixed to the value fit when the particular $\tilde{ℰ}$ was the test environment, and $f^{{ℰ}^{*}}$ is fit.

In practice, we assume that head direction uncertainty is small (because salient orientation cues were available in all experiments) and that tactile reliability is high, so we treat *h* as observed. Therefore, $f^{{ℰ}^{*}}$ and $f^{\tilde{ℰ}}$ depend on *h*, but we only need to compute the posterior over the location *ℓ*.

Also, for simplicity of optimization, we assume the limit of an infinite-sized neural population. (Note that this contrasts with our assumption of a finite retinotopic neural population (Eq. 12), where we used the Poisson noise as a proxy for noise and ambiguity inherent in the egocentric visual input.) This assumption allows us to express the right hand side of Eq. 17 with $f^{{ℰ}^{*}}$ instead of *r*:

(18) $$\alpha^{\prime }\sum_{i}\log\text{P}\left(r_{i}|\ell ;\;f_{i}^{\tilde{ℰ}}(z)\right)=\alpha^{\prime }\sum_{i}\sum_{j=1}^{N_{i}}\log\text{P}(r_{j}|\ell ;\;f_{i}{\tilde{ℰ}}_{\left(z)\right)}$$

(19) =α′∑i∑j=1Ni[rjTlog(fiℰ(z)T)−fiℰ(z)T−log(fiT)!︸⊥z]

(20) $$\log P^{\text{g}}(\ell ;\;z,\;\Theta )=\sum_{i}\alpha_{i}\left[f_{i}^{{ℰ}^{*}}(z)T\log\left(f_{i}^{\tilde{ℰ}}(z)T\right)-f_{i}^{\tilde{ℰ}}(z)T\right]+\underset{⊥z}{\underset{︸}{\ldots }}$$

where *N*_*i*_ is the number of neurons with the same tuning curve. We suppress the Poisson variability and use $f_{i}^{\varepsilon^{*}}$ in place of *r*_*i*_, by setting the population size *N*_*i*_ → ∞ and setting the contribution of an individual neuron *α*′ := *α*_*i*_/*N*_*i*_ → +0 while keeping *α*_*i*_ finite. For simplicity, we treat the *N*_*i*_ neurons that share the same tuning curve $f_{i}^{\varepsilon^{*}}$ as a single deterministic neuron *i* with a continuous firing rate $f_{i}^{\varepsilon^{*}}$ from here. We then fit ***α*** := {*α*_*i*_}, to account for the difference between the contribution of each neuron to the decoded neural posterior. Since our hypothesis is that warping is the dominant factor determining the posterior, we constrain *α*_*i*_ to be the same for all neurons within a grid module. We denote all parameters controlling the neural posterior together as **Θ** := (**Θ**^w^, ***α***). We fix *T* = 1 for simplicity.

To fit the warping, we use gradient descent to find:

(21) $$\hat{\Theta }=\underset{\Theta }{\mathrm{argmin}}\;{ℒ}^{\text{fit}}+{ℒ}^{\text{reg}}$$

where

(22) $${ℒ}^{\text{fit}}:={\langle {\text{D}}_{\text{KL}}\left[{\text{P}}_{t}^{\text{b}}\|P_{r_{t}}^{\text{g}}\right]\rangle }_{t}$$

is the divergence of the neural posterior from the ideal observer’s posterior, averaged across time. ${ℒ}^{\text{reg}}$ is a regularization loss that penalizes deviation of the angle between neighboring nodes of the mesh from 90°. It is defined as:

(23) $${ℒ}^{\text{reg}}:=w^{\text{reg}}\sum {\left(\frac{1\;-\;\sin\vartheta }{2}\right)}^{\beta }$$

Here, *θ* is an angle subtended by neighboring points in the mesh controlling the warp, *w*^reg^ and *β* are hyperparameters that control the weight and stiffness of the regularization, respectively.

It turns out that we can further simplify the calculation of the ${ℒ}^{\text{fit}}$, because it is equivalent to minimizing the divergence of the posterior (in our case, defined over believed location $\tilde{\ell }$), given a state that parameterizes the neural firing rate (in our case, **z**), *averaged across time*:

(24) $${\langle {\text{D}}_{\text{KL}}\left[{\text{P}}^{\text{b}}\left(\tilde{\ell };\;z_{t},\;t\right)\|P^{\text{g}}\left(\tilde{\ell };\;z_{t},\;\Theta \right)\right]\rangle }_{t}=\frac{1}{T}\sum_{t}\sum_{\tilde{\ell }}{\text{P}}^{\text{b}}\left(\tilde{\ell };\;z_{t},\;t\right)\left(\log{\text{P}}^{\text{b}}\left(\tilde{\ell };\;z_{t},\;t\right)-\log P^{\text{g}}\left(\tilde{\ell };\;z_{t},\;\Theta \right)\right)$$

(25) $$\;=C-\frac{1}{T}\sum_{z}\sum_{\tilde{\ell }}\log P^{\text{g}}(\tilde{\ell };\;z,\;\Theta )\cdot \sum_{t:z_{t}=z}{\text{P}}^{\text{b}}\left(\tilde{\ell };\;z_{t},\;t\right)$$

(26) $$\;=C-\frac{1}{T}\sum_{z}\sum_{\tilde{\ell }}\log P^{\text{g}}(\tilde{\ell };\;z,\;\Theta )M(z)\bar{{\text{P}}^{\text{b}}}(\tilde{\ell };\;z)$$

Here, *C* is a constant term that is independent of **Θ**, *M*(**z**) := |{*t* : **z**_*t*_ = **z**}| is the number of visits to **z**, and $\bar{{\text{P}}^{\text{b}}}(z):=\frac{1}{M(z)}\sum_{t:z_{t}=z}{\text{P}}^{\text{b}}\left(\tilde{\ell };\;z_{t},\;t\right)$ is the ideal observer’s posterior at **z** averaged across time, which is also independent of **Θ**. These two terms can be pre-computed, which greatly accelerates the optimization of **Θ** when |{**z**}| ≪ *T*, as is the case for our simulations.

To gauge the quality of the fit, we show optimized D_KL_ along with controls in Fig. 1e, which are defined as follows:

(27) $${\text{D}}_{\text{KL}}^{\text{optim}}:={\langle {\text{D}}_{\text{KL}}\left[{\text{P}}^{\text{b}}\left(\tilde{\ell };\;z_{t},\;t\right)\|P^{\text{g}}\left(\tilde{\ell };\;z_{t},\;\hat{\Theta }\right)\right]\rangle }_{t}$$

(28) $${\text{D}}_{\text{KL}}^{\text{within}}:=\frac{1}{\sum_{z^{\prime }}M\left(z^{\prime }\right)}\sum_{z^{\prime }}M\left(z^{\prime }\right){\text{D}}_{\text{KL}}\left[{\langle {\text{P}}^{\text{b}}\left(\tilde{\ell };\;z_{t}=z^{\prime }\right)\rangle }_{t}\|{\langle P^{\text{g}}\left(\tilde{\ell };\;z_{t}=z^{\prime },\hat{\Theta }\right)\rangle }_{t}\right]$$

(29) $${\text{D}}_{\text{KL}}^{\text{reg}}:={\langle {\text{D}}_{\text{KL}}\left[{\text{P}}^{\text{b}}\left(\tilde{\ell };\;z_{t},\;t\right)\|P^{\text{g}}\left(\tilde{\ell };\;z_{t},\;\Theta^{\text{reg}}\right)\right]\rangle }_{t}$$

(30) $${\text{D}}_{\text{KL}}^{\text{shuffle}}:={\langle {\text{D}}_{\text{KL}}[{\text{P}}^{\text{b}}\left(\tilde{l};\;z_{\tilde{t}(t)},\;\tilde{t}(t)\right)\|P^{\text{g}}\left(\tilde{l};\;z_{t},\;\hat{\Theta }\right)]\rangle }_{t}$$

where **Θ**^reg^ is the parameter of the warping that gives regular grid fields, and $\tilde{t}(t)$ is the *t*-th time step after shuffling.

For results in Fig. 4d, we only fit $f^{{ℰ}^{*}}$ while fixing $f^{\tilde{ℰ}}$ to be regular grid fields. This was because the familiar environment was always a square, in which the effect of warping was small.

### Homing task

#### Overview

Homing experiments^5,8,9^ consisted of a learning phase followed by a test phase. In the learning phase, subjects navigated to a visible target location. In the test phase, subjects had to navigate back to the hidden target location from memory, starting from a designated location that differed from the target location. In Ref. 9, subjects had to follow a designated path (that was randomly chosen by the computer program every trial) before they navigate back to the hidden target location.

In both phases, at each time step, a control signal **u** is chosen among four options: staying still, moving forward, turning right, and turning left. Formally:

(31) $$u:=\left(u^{\text{x}},\;u^{\theta }\right)$$

(32) ∈𝒞

(33) $$\;:=\left\{u^{0},\ldots ,u^{C}\right\}$$

(34) :={(0,0),(Δℓ,0),(Δℓ,−Δθ),(Δℓ,+Δθ)}

#### Learning phase

We modeled the observer to be from the starting location toward the true target location in as straight as possible trajectory at each moment (or following a designated path^9^). The control signals are sampled passively, i.e., without regard to the observer’s beliefs. If there are more than two possible such control signals at a given moment, one is chosen at random. When the observer arrives at the true target location, its belief about its location is stored as the mean believed target location $\bar{\ell }$, given all observations **o** and control signals **u** (which include translation and rotation):

(35) $${\tilde{\text{P}}}^{\text{learn}}\left({\tilde{\ell }}^{\text{learn}}\right)\propto \text{P}{\left({\tilde{\ell }}^{\text{learn}}|o_{0:T^{\text{learn}}}^{\text{learn}},\;u_{0:T^{\text{learn}}},\;\tilde{ℰ}\right)}^{\beta_{\text{learn}}}$$

(36) $$\;\bar{\ell }=\text{E}\left({\tilde{\ell }}^{\text{learn}}\right)$$

where 0 < *β*_learn_ ≤ 1 is the inverse temperature parameter that models forgetting (the lower, the more forgetting).

#### Test phase: position estimation

The observer chooses its own control signal. For this, it first computes the counterfactual expected value from executing each candidate control:

(37) $$v_{j}:=\sum_{{\tilde{\ell }}^{\text{learn}}}\sum_{{\tilde{\ell }}^{\text{test}}}\text{v}\left({\tilde{\ell }}^{\text{learn}},\;{\tilde{\ell }}^{\text{test}}\right){\tilde{P}}^{\text{learn}}\left({\tilde{\ell }}^{\text{learn}}\right){\tilde{\text{P}}}^{j}\left({\tilde{\ell }}^{\text{test}}\right)$$

where the value is defined to be the negative squared Euclidean distance:

(38) $$v\left({\tilde{\ell }}^{\text{learn}},\;{\tilde{\ell }}^{\text{test}}\right):=-{\left\|{\tilde{\ell }}^{\text{learn}}-{\tilde{\ell }}^{\text{test}}\right\|}_{2}^{2}$$

The counterfactual location posterior when staying is defined to be the same as the current posterior:

(39) $${\tilde{\text{P}}}^{0}\left({\tilde{\ell }}^{\text{test}}\right):=\text{P}\left({\tilde{\ell }}^{\text{test}}|o_{0:t}^{\text{test}},\;u_{0:t}^{\text{test}},\;\tilde{ℰ}\right)$$

In general, the counterfactual location distribution after executing the *j*-th candidate control **u**^*j*^ is defined in the believed environment space:

(40) $${\tilde{\text{P}}}^{j}\left({\tilde{\ell }}^{\text{test}}\right):=\text{P}\left({\tilde{\ell }}^{\text{test}}|o_{0:t}^{\text{test}},\;u_{0:t}^{\text{test}},\;u^{j},\;\tilde{ℰ}\right)$$

with a temperature parameter $\sigma_{\text{v}}^{2}$, which effectively scales the distance in Eq. 38:

(41) $$\text{P}\left(u_{t}^{\text{test}}=u^{j}\right)\propto e^{{\text{v}}_{j}/\sigma_{v}^{2}}$$

The final response is made when the observer chooses to stay, i.e., $u_{t}^{\text{test}}=u^{0}$, and the observer’s true location at that time is recorded as the responded location.

#### Test phase: distance estimation

In addition to the homing task, subjects in Ref. 5 also performed a distance estimation task. In this task, subjects were asked to indicate the distance between two target objects they have seen during the learning phase. During the test phase, subjects were asked to walk the distance they want to indicate in a virtual circular enclosure (to which they were previously familiarized). Specifically, they were asked to walk the same distance as the distance between a pair of objects, starting from a root of a static visual arrow placed randomly in the familiar circular environment, following the direction of the arrow, which always pointed to the center.

In our simulation, the observer is driven from a random starting location toward the root of the arrow in as straight as possible trajectory at each moment, until it arrives at the root (similar to the learning phase of the homing task Eq. 35). The observer starts this trajectory with a uniform prior belief over locations and heading directions, and ends it with a distribution of believed position of the root $\text{P}\left({\tilde{\ell }}_{\text{root}}\right)$. Note that, since the observer continues onto the estimation as soon as it reaches the root of the arrow, we assume it does not forget the position of the root (i.e., retains the full posterior without exponentiation by *β*_learn_, unlike Eq. 35 and Eq. 36).

At each time step from then on, the observer decides whether to walk another step in the direction of the arrow or stay, using the counterfactual value of the predicted location after each action (walk or stay), as in the test phase of the homing task Eq. 37–Eq. 41. Here, the value $v_{u}^{\text{est}}$ of a control signal *u*_*t*_ = *u* ∈ {walk, stay} is defined as the negative expected squared difference between the predicted distance from the root of the arrow and the believed distance between the given pair of objects (*j*, *k*):

(42) $$v_{u}^{\text{est}\;}=\sum_{\ell }\sum_{\ell^{\prime }}\tilde{\text{P}}\left({\tilde{\ell }}_{\text{root}}=\ell \right){\tilde{\text{P}}}^{u}\left(\ell^{\prime }|{\tilde{\ell }}_{\text{root}}=\ell ,\;u_{0:t-1},\;o_{0:t-1}\right)v^{\text{est}}\left({\left\|\ell -\ell^{\prime }\right\|}_{2}\right)$$

(43) $${\text{v}}^{\text{est}}\left({\left\|\ell -\ell^{\prime }\right\|}_{2}\right)=-{\left({\left\|\ell -\ell^{\prime }\right\|}_{2}-{\left\|{\bar{\ell }}_{j}-{\bar{\ell }}_{k}\right\|}_{2}\right)}^{2}$$

Then the choice is made based on the softmax over *u*, with the temperature parameter *σ*_v_, which effectively scales the difference between distances in Eq. 43, as it does in the homing task (Eq. 41). The estimation trial ends when the observer chooses to stay, and the observer’s true distance from the root of the arrow is recorded as the responded distance.

## Supplementary Material

Supplement 1

## Figures and Tables

**Table 1 |:** Choice of experimental tasks.

Environmental geometry	Behavioral (Human)	Neural (Rodent)
Square vs. trapezoid	Bellmund et al. 2020^5^	Krupic et al. 2015^6^
Expanded & compressed rectangles	Chen et al. 2015^9^, Hartley et al. 2004^8^, Keinath et al. 2020^12^	Barry et al. 2007^10^, Keinath et al. 2018^11^
