# Supplementary material for: Spatial uncertainty and environmental geometry in navigation
Source: bioRxiv. 2023 Feb 1:2023.01.30.526278. Preprint. [Version 1] doi: 10.1101/2023.01.30.526278 (PMC9915518; doi:10.1101/2023.01.30.526278)
Supplement: Supplement 1 [file NIHPP2023.01.30.526278v1-supplement-1.pdf]

bioRxiv preprint doi: https://doi.org/10.1101/2023.01.30.526278; this version posted February 1, 2023. The copyright holder for this preprint (which was not certified by peer review) is the author/funder, who has granted bioRxiv a license to display the preprint in perpetuity. It is made available under aCC-BY 4.0 International license.

Supplementary Material

Visual uncertainty

A point landmark and a single neuron

While all results are obtained from numerical simulation using Eq. 11, we also derived simple analytic relation between Fisher information and the environmental geometry to gain analytic insights, and checked its correspondence with the numerical simulation (Fig. 2a). Here we start by deriving the Fisher information I^{ℓ}_{ϵ} of the apparent position $\tilde{\epsilon}$ about the true distance ℓ along an axis of interest, assuming that the axis of interest is the same as the optical axis of a pinhole projection (the line of sight of our ideal observer). We further assume that the distance of the point landmark from the axis, ϵ, as well as blur σ (on the image plane at a focal length of 1 without loss of generality) is known, which gives the distribution of the apparent position $\tilde{\epsilon} \sim \mathcal{N}(\epsilon', \sigma)$ with $\epsilon' := \epsilon/\ell$. Then:

$$I^{\ell}_{\epsilon}(\ell) = \left\langle -\frac{\partial^2}{\partial \ell^2} \ln \mathrm{p}(\tilde{\epsilon} \mid \ell) \right\rangle_{\tilde{\epsilon}} \tag{S1}$$

$$= -\int_{-\infty}^{\infty} d\tilde{\epsilon}\, \mathrm{p}(\tilde{\epsilon} \mid \ell) \frac{\partial^2}{\partial \ell^2} \ln \mathrm{p}(\tilde{\epsilon} \mid \ell) \tag{S2}$$

$$= \frac{1}{2} C \int_{-\infty}^{\infty} d\tilde{\epsilon}\, e^{-\frac{1}{2}\left(\frac{\tilde{\epsilon}-\epsilon'}{\sigma}\right)^2} \frac{\partial^2}{\partial \ell^2} \left(\frac{\tilde{\epsilon}-\epsilon'}{\sigma}\right)^2 \tag{S3}$$

where $C = \frac{1}{\sigma\sqrt{2\pi}}$ is the normalization factor. The partial derivative evaluates to:

$$\frac{\partial^2}{\partial \ell^2} \left(\frac{\tilde{\epsilon}-\epsilon'}{\sigma}\right)^2 = 2\frac{\partial}{\partial \ell} \left(\frac{\tilde{\epsilon}-\epsilon\ell^{-1}}{\sigma}\right) \left(\frac{\epsilon}{\sigma}\ell^{-2}\right) \tag{S4}$$

$$= \frac{2}{\sigma^2} \frac{\partial}{\partial \ell} \left(\tilde{\epsilon}\epsilon\ell^{-2} - \epsilon^2\ell^{-3}\right) \tag{S5}$$

$$= \frac{2}{\sigma^2} \left(-2\tilde{\epsilon}\epsilon\ell^{-3} + 3\epsilon^2\ell^{-4}\right) \tag{S6}$$

$$= \underbrace{\frac{2}{\sigma^2}\left(-2\epsilon\ell^{-3}\right)}_{-4w\ell^{-3}/\sigma^2} \left(\tilde{\epsilon} - \epsilon' + \epsilon' + \underbrace{\frac{3\epsilon^2\ell^{-4}}{-2\epsilon\ell^{-3}}}_{-\frac{1}{2}\epsilon'}\right) \tag{S7}$$

Substituting back to Eq. S3:

$$I^{\ell}_{\epsilon}(\ell) = \frac{1}{2} C \left(-\frac{4\epsilon\ell^{-3}}{\sigma^2}\right) \int_{-\infty}^{\infty} d\tilde{\epsilon}\, e^{-\frac{1}{2}\left(\frac{\tilde{\epsilon}-\epsilon'}{\sigma}\right)^2} \left(\left(\tilde{\epsilon}-\epsilon'\right) - \frac{1}{2}\epsilon'\right) \tag{S8}$$

To evaluate the integral, note that:

$$\frac{\partial}{\partial \tilde{\epsilon}} e^{-\frac{1}{2}\left(\frac{\tilde{\epsilon}-\epsilon'}{\sigma}\right)^2} = -\frac{1}{\sigma}\left(\frac{\tilde{\epsilon}-\epsilon'}{\sigma}\right) e^{-\frac{1}{2}\left(\frac{\tilde{\epsilon}-\epsilon'}{\sigma}\right)^2} \tag{S9}$$

$$= -\frac{1}{\sigma^2}\left(\tilde{\epsilon}-\epsilon'\right) e^{-\frac{1}{2}\left(\frac{\tilde{\epsilon}-\epsilon'}{\sigma}\right)^2} \tag{S10}$$

Substituting back to Eq. S8:

$$I^{\ell}_{\epsilon}(\ell) = \frac{1}{2} C \left(-\frac{4\epsilon\ell^{-3}}{\sigma^2}\right) \left(-\sigma^2 \underbrace{\left[e^{-\frac{1}{2}\left(\frac{\tilde{\epsilon}-\epsilon'}{\sigma}\right)^2}\right]_{\tilde{\epsilon}=-\infty}^{\infty}}_{=0} - \frac{1}{2}\epsilon' \underbrace{\int_{-\infty}^{\infty} d\tilde{\epsilon}\, e^{-\frac{1}{2}\left(\frac{\tilde{\epsilon}-\epsilon'}{\sigma^2}\right)^2}}_{=1/C}\right) \tag{S11}$$

$$= \frac{\epsilon^2}{\sigma^2\ell^4} \tag{S12}$$

The result's unit is $(\text{length})^{-2}$, as it should be from Eq. S1, where $\frac{\partial^2}{\partial \ell^2}$ (whose unit is $(\text{length})^{-2}$) is applied to $((\tilde{\epsilon}-\epsilon')/\sigma)^2$, which is dimensionless since $\tilde{\epsilon}$, ϵ', and σ are dimensionless ratios between lengths (e.g., $\epsilon' := \epsilon/\ell$).

bioRxiv preprint doi: https://doi.org/10.1101/2023.01.30.526278; this version posted February 1, 2023. The copyright holder for this preprint (which was not certified by peer review) is the author/funder, who has granted bioRxiv a license to display the preprint in perpetuity. It is made available under aCC-BY 4.0 International license.

An array of Poissonian neurons

Now we derive the Fisher information $I_v^\ell(\ell)$ about ℓ given the firing rates $\mathbf{r}$ of an array of N neurons with independent Poisson spikes, which is defined as:

$$I_v^\ell(\ell) := -\int_{\mathbf{r}} d\mathbf{r}\, \mathrm{p}(\mathbf{r} \mid \ell) \frac{\partial^2}{\partial \ell^2} \ln \mathrm{p}(\mathbf{r} \mid \ell) \tag{S13}$$

While the results of this step has been known[32], we describe them here for completeness. Here, our aim is to express $I_v^\ell(\ell)$ as a function of each neuron i's tuning curve $f_i(\ell)$, which is defined as the mean firing rate given the true stimulus ℓ, i.e.:

$$f_i(\ell) := \int_{r_i} dr_i\, \mathrm{p}(r_i \mid \ell) r_i \tag{S14}$$

First consider $\mathrm{p}(\mathbf{r} \mid \ell)$ in Eq. S13. Since we assume that each neuron's spikes follow independent Poisson distribution, the likelihood of observing a certain vector of firing rates $\mathbf{r}$ is simply:

$$\mathrm{p}(\mathbf{r} \mid \ell) = \prod_{i=1}^{N} \frac{(f_i(\ell)T)^{r_i T}}{(r_i T)!} \tag{S15}$$

where T is the duration during which the spikes are observed. Now we can evaluate the partial derivative in Eq. S13 using Eq. S15:

$$\frac{\partial^2}{\partial \ell^2} \ln \mathrm{p}(\mathbf{r} \mid \ell) = \frac{\partial^2}{\partial \ell^2} \ln \left(\prod_{i=1}^{N} \frac{(f_i(\ell)T)^{r_i T}}{(r_i T)!} \right) \tag{S16}$$

$$= \frac{\partial^2}{\partial \ell^2} \left(\sum_{i=1}^{N} (r_i T \ln (f_i(\ell)T) - f_i(\ell)T - \ln(r_i T)!) \right) \tag{S17}$$

$$= \frac{\partial}{\partial \ell} \left(T \sum_{i=1}^{N} \left(\frac{T f_i'(\ell)}{f_i(\ell)T} r_i - f_i'(\ell) \right) \right) \tag{S18}$$

$$= T \sum_{i=1}^{N} \left(\left(\frac{f_i''(\ell)}{f_i(\ell)} - \frac{f_i'(\ell)^2}{f_i(\ell)^2} \right) r_i - f_i''(\ell) \right) \tag{S19}$$

Finally, substitute Eq. S19 back into Eq. S13 and simplify using Eq. S14:

$$I_v^\ell(\ell) = -\int_{\mathbf{r}} d\mathbf{r}\, \mathrm{p}(\mathbf{r} \mid \ell) T \sum_{i=1}^{N} \left(\left(\frac{f_i''(\ell)}{f_i(\ell)} - \frac{f_i'(\ell)^2}{f_i(\ell)^2} \right) \underbrace{r_i}_{\text{use Eq. S14}} - f_i''(\ell) \right) \tag{S20}$$

$$= -T \sum_{i=1}^{N} \left(\left(-\frac{f_i'(\ell)^2}{f_i(\ell)^{\cancel{2}}} \cancel{f_i(\ell)} + \frac{\cancel{f_i''(\ell)}}{\cancel{f_i(\ell)}} \cancel{f_i(\ell)} - \cancel{f_i''(\ell)} \right) \right) \tag{S21}$$

$$= T \sum_{i=1}^{N} \frac{f_i'(\ell)^2}{f_i(\ell)} \tag{S22}$$

Therefore, as aimed, we expressed $I_v^\ell(\ell)$ as a function of $f_i(\ell)$.

A retinal image

We are finally ready to derive the Fisher information $I_v^\ell(\ell)$ about the 3D distance ℓ from neurons with a uniform 2D retinotopic array of tuning curves. Assume a Gaussian tuning curve

$$f_i(\ell) := r_{\max} e^{-\frac{1}{2}\left(\frac{\epsilon_i - \epsilon/\ell}{\sigma}\right)^2} \tag{S23}$$

then

$$f_i'(\ell) = -\frac{1}{2} \cdot 2 \left(\frac{\epsilon}{\sigma} \ell^{-2} \right) \left(\frac{\epsilon_i}{\sigma} - \frac{\epsilon}{\sigma} \ell^{-1} \right) f_i(\ell) \tag{S24}$$

$$= -\frac{\epsilon}{\sigma^2 \ell^2} (\epsilon_i - \epsilon \ell^{-1}) f_i(\ell) \tag{S25}$$

bioRxiv preprint doi: https://doi.org/10.1101/2023.01.30.526278; this version posted February 1, 2023. The copyright holder for this preprint (which was not certified by peer review) is the author/funder, who has granted bioRxiv a license to display the preprint in perpetuity. It is made available under aCC-BY 4.0 International license.

Substitute Eq. S23 and Eq. S25 into Eq. S22:

$$I_v^\ell(\ell) = \frac{\epsilon^2 T}{\ell^4} \sum_{i=1}^{N} \left(\frac{(\epsilon_i - \epsilon\ell^{-1})^2}{\sigma^4} f_i(\ell) \right) \tag{S26}$$

Compare this with Ref. 32, Eqs. 3.46-48 to approximate the sum with an integral to obtain:

$$I_v^\ell(\ell) = \frac{\epsilon^2}{\ell^4} \cdot \frac{(2\pi)^{D/2} \rho \sigma^{D-2} r_{\max} T}{D} \tag{S27}$$

$$= \pi \rho r_{\max} T \cdot \frac{\epsilon^2}{\ell^4} \tag{S28}$$

where ρ is the number of neurons in a unit range of $\epsilon' := \epsilon/\ell$, and D is the number of dimensions of the space where the neurons are arranged, which is 2 for us. This expression is used for the lines in Fig. 2a, and its simplified form is presented as Eq. 1. Note that here we used ℓ instead of d to avoid confusion during integration.

Intriguingly, as explained in Ref. 32, the Fisher information is independent of the width of the tuning curve σ, because the number of neurons responding to the stimulus scales with $\rho\sigma^D$. The difference from Ref. 32 is the factor of ϵ^2/ℓ^4, which arises because our quantity of interest ℓ is inversely related to the center of the tuning curve ϵ/ℓ (rather than linearly related, which would have given the center of ℓ). Note that the resulting expression for $I_v^\ell(\ell)$ has the same unit as $I_\epsilon^\ell(\ell)$, and is proportional to it without the σ term, scaled with the number of spikes, $\rho r_{\max} T$.

Bias in distance estimation – analytic intuition

Our numerical simulation replicated different biases in distance estimation between environments and between sides of the trapezoid (Fig. 2d, bottom). Here, we provide analytic intuition for the results with a simplified version of the ideal observer model. We derive a relation that explains how strongly the bias in distance estimation along a given axis (x or y) depends on the (half of the) sizes of the environment along that axis ('depth', L) and the axis orthogonal to it ('eccentricity', ϵ). Then we use this relation to show how the relative sizes of the environments and its sides (Fig. S1) translate into the difference in biases.

First, we express $\hat{\ell}$, the estimated location, obtained with Bayesian inference after Laplace approximation of the prior and the visual likelihood, as a function of L and ϵ. Here, without loss of generality, we adopt a convention where $\hat{\ell} = 0$ means that the estimated location matches the mean prior location:

$$\hat{\ell} = f(\ell)\ell \qquad 0 < \ell < L \tag{S29}$$

$$\text{where } f(\ell) := \frac{1}{1 + g(\ell)} \qquad 0 < f(\ell) < 1 \tag{S30}$$

$$\text{and } g(\ell) := \frac{(L-\ell)^4}{\epsilon^2} \frac{\lambda}{L^2} \qquad 0 < g(\ell) \tag{S31}$$

Here, $L - \ell$ is the distance to the wall in front, $\frac{\epsilon^2}{(L-\ell)^4}$ is the precision of the likelihood (following Eq. S28), $\frac{\lambda}{L^2}$ is the precision of the prior (which is lower in a larger environment), where λ is a free parameter that indicates the relative informativeness of the prior compared to the likelihood.

Then, we express how much g depends on L vs. ϵ in preparation for a later comparison on how much the estimated location $\hat{\ell}$ depends on them:

$$\frac{\partial g}{\partial L} = 2\frac{g(\ell)}{L-\ell}\left(2 - \frac{L-\ell}{L}\right) \tag{S32}$$

$$\frac{\partial g}{\partial \epsilon} = -\frac{2}{\epsilon} g(\ell) \tag{S33}$$

which means that, since $L - \ell \in [0, L]$ and $2 - \frac{L-\ell}{L} \in [0, 2]$, when $L = \epsilon$:

$$\left|\frac{\partial g}{\partial L}\right| > \left|\frac{\partial g}{\partial \epsilon}\right| \tag{S34}$$

bioRxiv preprint doi: https://doi.org/10.1101/2023.01.30.526278; this version posted February 1, 2023. The copyright holder for this preprint (which was not certified by peer review) is the author/funder, who has granted bioRxiv a license to display the preprint in perpetuity. It is made available under aCC-BY 4.0 International license.

In other words, g changes more with L than with ϵ. Now we express the estimated distance $\hat{d}$ between two target locations ℓ_1 and ℓ_2 as a function of f, and in turn, of g:

$$d = \ell_1 - \ell_2 \qquad \ell_1 > \ell_2 \Rightarrow f(\ell_1) > f(\ell_2) \tag{S35}$$

$$\hat{d} = \hat{\ell}_1 - \hat{\ell}_2 \tag{S36}$$

$$= f(\ell_1)\ell_1 - f(\ell_2)\ell_2 \tag{S37}$$

Then we compare how the change in L vs. ϵ affects $\hat{d}$ using the relations derived above:

$$\frac{\partial \hat{d}}{\partial g} = -f^2(\ell_1)\ell_1 + f^2(\ell_2)\ell_2 \tag{S38}$$

$$\frac{\partial \hat{d}}{\partial L} = \frac{\partial g}{\partial L}\frac{\partial \hat{d}}{\partial g} < 0 \tag{S39}$$

$$\frac{\partial \hat{d}}{\partial \epsilon} = \frac{\partial g}{\partial \epsilon}\frac{\partial \hat{d}}{\partial g} > 0 \tag{S40}$$

In words, the estimated distance between two target locations ℓ_1 and ℓ_2 is shorter when the environment is deeper (when the wall is farther and visual likelihood is less precise) due to stronger regression to mean, and longer when the environment is wider due to weaker regression to mean. From Eq. S34, the absolute values of the above two quantities can be compared as:

$$\left|\frac{\partial \hat{d}}{\partial L}\right| > \left|\frac{\partial \hat{d}}{\partial \epsilon}\right| \tag{S41}$$

Therefore, the estimated distance relies more on the depth of the environment than on its width.

Finally, we use Eq. S41 to compare the estimated distances in square vs. trapezoid, and in the broad vs. narrow sides of the trapezoid, which we denote as $\hat{S}$ (square), $\hat{T}$ (trapezoid), $\hat{B}$ (broad side of the trapezoid), and $\hat{N}$ (narrow side of the trapezoid). We further denote the size of the environments and the halves of the trapezoid as $X_.$ and $Y_.$ as shown in Fig. S1.

Using these relations, we begin by comparing the estimated distance along the x axis between the square and trapezoid environments. Here, X_S and X_T are the depths. (To be exact, the prior mean x location of the trapezoid is off center, but we ignore it for simplicity and use X_T for L, since the following holds as long as $X_S \ll X_T$.) Since $X_S \ll X_T$, we can infer that $\hat{S} \gg \hat{T}$ (larger depth results in shorter estimated distance; Eq. S39). To disambiguate this comparison with others, we denote it as $\hat{S}^X_L \gg \hat{T}^X_L$, which means we used X_S and X_T as the depth (L) of the environment.

Likewise, we can compare the widths (ϵ) of the environments as $Y_S < Y_T$, from which we infer that $\hat{S}^Y_\epsilon > \hat{T}^Y_\epsilon$ (larger width results in longer estimated distance; Eq. S40) using the same convention as above.

We can also compare the estimated distance along the y axis. Then Y_S and Y_T would be the depths, and X_S and X_T the widths. We can summarize the comparisons along the x and y axes as:

$$\text{square vs. trapezoid}\begin{cases}\text{along x axis}\begin{cases}X_S \ll X_T & \Rightarrow \quad \hat{S}^X_L \gg \hat{T}^X_L\\ Y_S > Y_T & \Rightarrow \quad \hat{S}^Y_\epsilon > \hat{T}^Y_\epsilon\end{cases}\\ \\ \text{along y axis}\begin{cases}Y_S > Y_T & \Rightarrow \quad \hat{S}^Y_L < \hat{T}^Y_L\\ X_S \ll X_T & \Rightarrow \quad \hat{S}^X_\epsilon \ll \hat{T}^X_\epsilon\end{cases}\end{cases} \tag{S42}$$

Thus, the estimated distance in the square should be longer along the x axis, while it should be shorter along the y axis. To resolve this conflict, we compare the relative importance of the two axes. According to Eq. S41, the influence of the depth should dominate the influence of the width when $L = \epsilon$. This allows us to compare, e.g., $|\hat{S}^X_L - \hat{T}^X_L|$ and $|\hat{S}^X_\epsilon - \hat{T}^X_\epsilon|$, where the same set of sizes (X_S and X_T) were used. This, combined with the relative size difference of the two environments along the two axes means that:

$$\left.\begin{array}{lll}|\hat{S}^X_L - \hat{T}^X_L| > |\hat{S}^X_\epsilon - \hat{T}^X_\epsilon| & \Rightarrow & \hat{S} \gg \hat{T}\\ |\hat{S}^Y_L - \hat{T}^Y_L| > |\hat{S}^Y_\epsilon - \hat{T}^Y_\epsilon| & \Rightarrow & \hat{S} < \hat{T}\end{array}\right\} \Rightarrow \quad \hat{S} > \hat{T} \tag{S43}$$

bioRxiv preprint doi: https://doi.org/10.1101/2023.01.30.526278; this version posted February 1, 2023. The copyright holder for this preprint (which was not certified by peer review) is the author/funder, who has granted bioRxiv a license to display the preprint in perpetuity. It is made available under aCC-BY 4.0 International license.

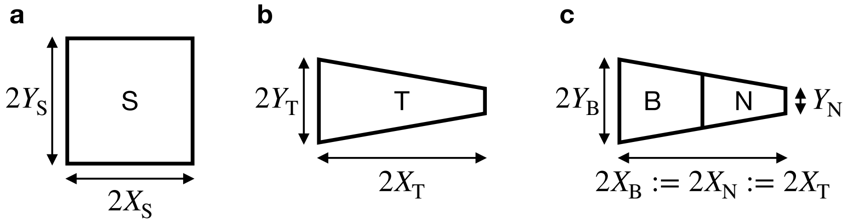

Fig. S1 | Symbols used to explain the analytic intuition for the bias in distance estimation. Schematics of the environments where their relative sizes roughly follow that of Ref. 5, where the distance estimation experiments are reported. **a.** Whole square. **b.** Whole trapezoid. **c.** Broad and narrow side of the trapezoid, split in half. Because the subject does not know whether they are in the broad or narrow half, their prior along the x axis covers the whole environment. Hence, we define $X_B := X_N := X_T$ so that any of them can substitute L from Eq. S29 to Eq. S41.

which agrees with the experimental results (Fig. 2d bottom, green vs. orange).

Likewise, we compare the broad vs. narrow sides of the trapezoid. Note that the two sides share the same size along the x axis (which is X_T) for the purpose of determining the precision of the prior and likelihood:

$$\text{broad vs. narrow side of the trapezoid} \begin{cases} \text{along x axis} \begin{cases} X_B = X_N = X_T & \Rightarrow \quad \hat{B}_L^X = \hat{N}_L^X \\ Y_B > Y_N & \Rightarrow \quad \hat{B}_\epsilon^Y > \hat{N}_\epsilon^Y \end{cases} \\ \text{along y axis} \begin{cases} Y_B > Y_N & \Rightarrow \quad \hat{B}_L^Y < \hat{N}_L^Y \\ X_B = X_N = X_T & \Rightarrow \quad \hat{B}_\epsilon^X = \hat{N}_\epsilon^X \end{cases} \end{cases} \tag{S44}$$

Again, we compare them with Eq. S41:

$$\left.\begin{array}{lll} |\hat{B}_L^X - \hat{N}_L^X| = |\hat{B}_\epsilon^X - \hat{N}_\epsilon^X| = 0 & \Rightarrow & \hat{B} = \hat{N} \\ |\hat{B}_L^Y - \hat{N}_L^Y| > |\hat{B}_\epsilon^Y - \hat{N}_\epsilon^Y| & \Rightarrow & \hat{B} < \hat{N} \end{array}\right\} \Rightarrow \quad \hat{B} < \hat{N} \tag{S45}$$

which, again, agrees with the experimental results (Fig. 2d bottom, light vs. dark orange). Therefore, we can see that the difference in the estimated distance (Eqs. S43 and S45) can be explained by the effect of the size of the environments on the precision of the prior and likelihood in Bayesian inference.

Neural tethering – analytic intuition

Our goal is to show that, if the ideal observer's posterior is tethered to the last wall approached, then a neural posterior, if it is encoded by tuning curves tethered to the last wall approached, also matches the ideal observer's posterior. Since tuning curves define mean firing rates, we first consider the mean posterior $\overline{\mathrm{P}}$, defined as the ideal observer posterior averaged over time:

$$\overline{\mathrm{P}}\left(\tilde{\boldsymbol{\ell}}|\boldsymbol{\ell},\theta,\mathcal{E}\right) := \left\langle \mathrm{P}\left(\tilde{\boldsymbol{\ell}}|\tilde{u}_{0:t},\mathbf{v}_{0:t},\mathcal{E}\right)\right\rangle_{t:(\boldsymbol{\ell}_t^*,\theta_t^*)=(\boldsymbol{\ell},\theta)} \tag{S46}$$

For simplicity, here we only consider the 1D location $\ell \in \mathcal{L} = [0, L]$ along the changed axis, and two opposite head directions along that axis, $\theta \in \Omega = \{0, \pi\}$, where $\theta = 0$ means the observer faces toward L. We also assume that the momentary posterior of the ideal observer matches its average posterior in either environment $\mathcal{E} \in \{\tilde{\mathcal{E}}, \mathcal{E}^*\}$, which allows us to parameterize the ideal observer posterior P^b with location, head direction, and environment, as follows:

$$\begin{aligned} \mathrm{P}^b\left(\tilde{\ell};\ell_t^*,\theta_t^*,\mathcal{E}\right) &:= \overline{\mathrm{P}}\left(\tilde{\ell}|\ell_t^*,\theta_t^*,\mathcal{E}\right) \\ &= \mathrm{P}\left(\tilde{\ell}|\tilde{u}_{0:t},\mathbf{v}_{0:t},\mathcal{E}\right) \quad \forall t \end{aligned} \tag{S47}$$

bioRxiv preprint doi: https://doi.org/10.1101/2023.01.30.526278; this version posted February 1, 2023. The copyright holder for this preprint (which was not certified by peer review) is the author/funder, who has granted bioRxiv a license to display the preprint in perpetuity. It is made available under aCC-BY 4.0 International license.

Simplifying the approximate patterns seen in behavioral data, assume that the ideal observer's belief $\mathrm{P^b}$ is tethered to the last wall in the new environment $\mathcal{E}^*$, and that the last wall is always behind the observer (i.e., when $\theta = 0$, the last wall is at 0, and when $\theta = \pi$, the last wall is at L^{new}). For $\theta^* = 0$, this is simply:

$$\underbrace{\mathrm{P^b}\left(\tilde{\ell};\ell^*,\theta^*=0,\mathcal{E}^*\right)}_{\mathrm{P^b_{new}}(\theta^*=0)} \propto \underbrace{\mathrm{P^b}\left(\tilde{\ell};\ell^*,\theta^*=0,\tilde{\mathcal{E}}\right)}_{\mathrm{P^b_{familiar}}(\theta^*=0)} \quad \forall\, \ell^* \in [0, L^{\text{new}}] \tag{S48}$$

We also assume an exact match between the ideal observer and neural posteriors in $\tilde{\mathcal{E}}$:

$$\underbrace{\mathrm{P^b}\left(\tilde{\ell};\ell^*,\theta^*,\tilde{\mathcal{E}}\right)}_{\mathrm{P^b_{familiar}}} = \underbrace{P^{\mathrm{g}}\left(\tilde{\ell}\,|\,\underbrace{f(\ell^*,\theta^*,\tilde{\mathcal{E}})}_{f_{\text{familiar}}}\right)}_{P^{\mathrm{g}}_{\text{familiar}}} \quad \forall\, (\ell^*,\theta^*) \tag{S49}$$

Note that we are considering the limit of a large neural population size, which gives $r_t = f(\ell_t^*,\theta_t^*,\mathcal{E})\,\forall t$, and the limit of good coverage of the environment by the observer's trajectory, which gives $\{(\ell_t^*,\theta_t^*)\} = \mathcal{L}\times\Theta$. Here, for simplicity, we assume $L^{\text{new}} < L^{\text{familiar}}$, i.e., that the observer is in a new environment $\mathcal{E}^*$ compressed compared to the familiar environment $\tilde{\mathcal{E}}$, such that tethered beliefs can cover the whole length of $\mathcal{E}^*$.

Then, having tethered tuning curve, i.e.,

$$\underbrace{f(\ell^*,\theta^*=0,\mathcal{E}^*)}_{f_{\text{new}}(\theta^*=0)} = \underbrace{f(\ell^*,\theta^*=0,\tilde{\mathcal{E}})}_{f_{\text{familiar}}(\theta^*=0)} \quad \forall\, \ell^* \in [0, L^{\text{new}}] \tag{S50}$$

which implies

$$\underbrace{P^{\mathrm{g}}\left(\tilde{\ell}\,|\,\underbrace{f(\ell^*,\theta^*=0,\tilde{\mathcal{E}})}_{f_{\text{familiar}}(\theta^*=0)}\right)}_{P^{\mathrm{g}}_{\text{familiar}}(\theta^*=0)} \propto \underbrace{P^{\mathrm{g}}\left(\tilde{\ell}\,|\,\underbrace{f(\ell^*,\theta^*=0,\mathcal{E}^*)}_{f_{\text{new}}(\theta^*=0)}\right)}_{P^{\mathrm{g}}_{\text{new}}(\theta^*=0)} \quad \forall\, \ell^* \in [0, L^{\text{new}}] \tag{S51}$$

is, by transitivity across Eqs. S48, S49 and S51, sufficient to give

$$\underbrace{P^{\mathrm{g}}\left(\tilde{\ell}\,|\,\underbrace{f(\ell^*,\theta^*=0,\mathcal{E}^*)}_{f_{\text{new}}(\theta^*=0)}\right)}_{P^{\mathrm{g}}_{\text{new}}(\theta^*=0)} = \underbrace{\mathrm{P^b}\left(\tilde{\ell};\ell^*,\theta^*=0,\mathcal{E}^*\right)}_{\mathrm{P^b_{new}}(\theta^*=0)} \quad \forall\, \ell^* \in [0, L^{\text{new}}] \tag{S52}$$

Also, in the limits of good coverage that we're assuming, it is also natural to assume mirror symmetry in $\tilde{\mathcal{E}}$, i.e.,

$$\underbrace{\mathrm{P^b}\left(\tilde{\ell};\ell^*,\theta^*=0,\tilde{\mathcal{E}}\right)}_{\mathrm{P^b_{familiar}}(\theta^*=0)} = \underbrace{\mathrm{P^b}\left(\tilde{\ell};L^{\text{familiar}}-\ell^*,\theta^*=\pi,\tilde{\mathcal{E}}\right)}_{\mathrm{P^b_{familiar}}(\theta^*=\pi)} \tag{S53}$$

and the same for the neural posterior and tuning curves. Then, by mirror symmetry, results analogous to Eq. S52 also hold when $\theta^* = \pi$. That is, tethering of the ideal observer's belief...

$$\underbrace{\mathrm{P^b}\left(\tilde{\ell};L^{\text{new}}-\ell^*,\theta^*=\pi,\mathcal{E}^*\right)}_{\mathrm{P^b_{new}}(\theta^*=\pi)} \propto \underbrace{\mathrm{P^b}\left(\tilde{\ell};L^{\text{familiar}}-\ell^*,\theta^*=\pi,\tilde{\mathcal{E}}\right)}_{\mathrm{P^b_{familiar}}(\theta^*=\pi)} \quad \forall\, \ell^* \in [0, L^{\text{new}}] \tag{S54}$$

... and tethering of tuning curves...

$$\underbrace{f(L^{\text{new}}-\ell^*,\theta^*=0,\mathcal{E}^*)}_{f_{\text{new}}(\theta^*=0)} = \underbrace{f(L^{\text{familiar}}-\ell^*,\theta^*=0,\tilde{\mathcal{E}})}_{f_{\text{familiar}}(\theta^*=0)} \quad \forall\, \ell^* \in [0, L^{\text{new}}] \tag{S55}$$

... which imply...

$$\underbrace{P^{\mathrm{g}}\left(\tilde{\ell}\,|\,\underbrace{f(L^{\text{familiar}}-\ell^*,\theta^*=\pi,\tilde{\mathcal{E}})}_{f_{\text{familiar}}(\theta^*=\pi)}\right)}_{P^{\mathrm{g}}_{\text{familiar}}(\theta^*=\pi)} \propto \underbrace{P^{\mathrm{g}}\left(\tilde{\ell}\,|\,\underbrace{f(L^{\text{new}}-\ell^*,\theta^*=\pi,\mathcal{E}^*)}_{f_{\text{new}}(\theta^*=\pi)}\right)}_{P^{\mathrm{g}}_{\text{new}}(\theta^*=\pi)} \quad \forall\, \ell^* \in [0, L^{\text{new}}] \tag{S56}$$

bioRxiv preprint doi: https://doi.org/10.1101/2023.01.30.526278; this version posted February 1, 2023. The copyright holder for this preprint (which was not certified by peer review) is the author/funder, who has granted bioRxiv a license to display the preprint in perpetuity. It is made available under aCC-BY 4.0 International license.

... are, by transitivity across Eqs. S49, S54 and S56, sufficient for the neural posterior to match the ideal observer's posterior given $\theta^* = \pi$:

$$\underbrace{P^{\mathrm{g}}\left(\tilde{\ell} | \underbrace{f(L^{\mathrm{new}} - \ell^*, \theta^* = 0, \mathcal{E}^*)}_{f_{\mathrm{new}}(\theta^*=\pi)}\right)}_{P^{\mathrm{g}}_{\mathrm{new}}(\theta^*=\pi)} = \underbrace{\mathrm{P}^{\mathrm{b}}\left(\tilde{\ell}; L^{\mathrm{new}} - \ell^*, \theta^* = \pi, \mathcal{E}^*\right)}_{\mathrm{P}^{\mathrm{b}}_{\mathrm{new}}(\theta^*=\pi)} \quad \forall\ \ell^* \in [0, L^{\mathrm{new}}] \tag{S57}$$

Therefore, tuning curves tethered to the last wall in $\mathcal{E}^*$ is sufficient for the neural likelihood to be tethered the same way, and when the likelihood is sharp enough, for the neural posterior to match the ideal observer posterior tethered to the last wall approached in either direction, as in Eqs. S52 and S57.
